# Innate immunity in the simplest animals – placozoans

**DOI:** 10.1186/s12864-018-5377-3

**Published:** 2019-01-05

**Authors:** Kai Kamm, Bernd Schierwater, Rob DeSalle

**Affiliations:** 1 ITZ Ecology and Evolution, University of Veterinary Medicine Hannover, Foundation, Bünteweg 17d, D-30559 Hannover, Germany; 2 Sackler Institute for Comparative Genomics and Division of Invertebrate Zoology, American Museum of Natural History, New York, NY USA; 3 Ecology and Evolutionary Biology, Yale University, New Haven, CT 06520 USA

**Keywords:** Innate immunity, Placozoa, *Trichoplax*, Symbiosis, Toll-like receptor pathway, NOD-like receptor pathway, Scavenger receptors, Fibrinogen-related proteins

## Abstract

**Background:**

Innate immunity provides the core recognition system in animals for preventing infection, but also plays an important role in managing the relationship between an animal host and its symbiont. Most of our knowledge about innate immunity stems from a few animal model systems, but substantial variation between metazoan phyla has been revealed by comparative genomic studies. The exploration of more taxa is still needed to better understand the evolution of immunity related mechanisms. Placozoans are morphologically the simplest organized metazoans and the association between these enigmatic animals and their rickettsial endosymbionts has recently been elucidated. Our analyses of the novel placozoan nuclear genome of *Trichoplax* sp. H2 and its associated rickettsial endosymbiont genome clearly pointed to a mutualistic and co-evolutionary relationship. This discovery raises the question of how the placozoan holobiont manages symbiosis and, conversely, how it defends against harmful microorganisms. In this study, we examined the annotated genome of *Trichoplax* sp. H2 for the presence of genes involved in innate immune recognition and downstream signaling.

**Results:**

A rich repertoire of genes belonging to the Toll-like and NOD-like receptor pathways, to scavenger receptors and to secreted fibrinogen-related domain genes was identified in the genome of *Trichoplax* sp. H2. Nevertheless, the innate immunity related pathways in placozoans deviate in several instances from well investigated vertebrates and invertebrates. While true Toll- and NOD-like receptors are absent, the presence of many genes of the downstream signaling cascade suggests at least primordial Toll-like receptor signaling in Placozoa. An abundance of scavenger receptors, fibrinogen-related domain genes and Apaf-1 genes clearly constitutes an expansion of the immunity related gene repertoire specific to Placozoa.

**Conclusions:**

The found wealth of immunity related genes present in Placozoa is surprising and quite striking in light of the extremely simple placozoan body plan and their sparse cell type makeup. Research is warranted to reveal how Placozoa utilize this immune repertoire to manage and maintain their associated microbiota as well as to fend-off pathogens.

**Electronic supplementary material:**

The online version of this article (10.1186/s12864-018-5377-3) contains supplementary material, which is available to authorized users.

## Background

The discrimination between self and non-self is crucial for defense against infection and thus for maintaining an organism’s genetic and somatic integrity. In animals this defense is accomplished by an innate immune system comprised of pattern recognition receptors (PRRs) specifically recognizing pathogen-associated molecular patterns (PAMPs) [1]. While vertebrates have evolved a highly sophisticated adaptive immune system, the evolutionary older innate immune system constitutes the main defense against novel invading pathogens in vertebrates and invertebrates and has also been described in lower metazoans like Cnidaria (e.g. [2]). In recent years, the exploration of genomic data from other non-model animals, such as lophotrochozoans or basal chordates, has further extended our understanding about the diversity and evolution of innate immunity components present in animals (e.g. [3–6]). In addition, it has been recognized that innate immunity plays a fundamental role in managing symbiotic interactions between microorganisms and animal hosts. Accordingly, it has been hypothesized that it has, at least in part, evolved to mediate a bidirectional cross-talk between both partners of a mutualistic relationship [7]. In this view a host’s innate immunity is able to discern, tolerate and promote beneficial microorganisms while simultaneously fending off pathogens. Well investigated examples for such cross-talks among metazoans are the freshwater polyp *Hydra*, which seems to “actively select and shape its bacterial community” [8], and the symbiosis between the bobtail squid *Euprymna* and the bioluminescent bacterium *Vibrio fisheri*, which is selected and maintained by the host through PRR signaling (reviewed in [7]).

Amongst the basal animal phyla (Placozoa, Porifera, Cnidaria and Ctenophora), placozoans (for review see [9, 10]) clearly exhibit the simplest organized body plan, comprising only six somatic cell types identified so far [11–13]. Potential intracellular bacterial symbionts have been posited in these animals since ultrastructural analyses of *Trichoplax adhaerens* revealed bacterial enclosures in a subpopulation of fiber cells and within developing oocytes [11, 14, 15]. These findings suggested that placozoans are able to control the density as well as the distribution of their endosymbionts and that these are propagated to the next generation via vegetative and sexual reproduction. In 2013 Driscoll et al. [16] were able to identify the placozoan endosymbiont. Previously overlooked bacterial sequences in the *Trichoplax adhaerens* reference genome [17] were identified as fragments of a rickettsial endosymbiont genome. Rickettsiales belong to the α-Proteobacteria and constitute an order of obligate intracellular bacteria that are incapable to grow and divide outside the host because they have lost many important metabolic pathways [18, 19].

Recently we sequenced and investigated the genome of the placozoan 16S haplotype H2 (*Trichoplax* sp. H2; [20]), a close relative of *Trichoplax adhaerens*, and the genome assembly also yielded the complete genome of a placozoan rickettsial endosymbiont, which is described in Kamm et al., (in prep.). The results of the bacterial genome analyses, in terms of genome size, gene content and composition of metabolic pathways, clearly point to a mutualistic relationship which raises questions about how it has been established and how it is maintained. Relevant to understanding the placozoan holobiont is, if *Trichoplax* possesses an innate immunity system at all, and how it is organized to recognize harmful microorganisms on the one hand and to interact with beneficial microorganisms on the other. The small number of cell types and lack of specialized cells in placozoans further raises interesting questions about how immunity works in these most simple animals.

Recognition of non-self molecular patterns by the innate immune system takes place either on the extracellular side, via secreted or membrane bound PRRs, or inside a cell via intracellular receptors. A well investigated component of extracellular recognition in vertebrates and invertebrates includes the Toll-like receptors (TLR) [21], while intracellular recognition seems mainly mediated by the nucleotide binding and oligomerization domain like receptors (NOD-like), the retinoic-acid inducible gene I like receptors (RIG-I-like) or the stimulator of interferon genes receptor (STING) in conjunction with the cytosolic DNA sensor cyclic GMP-AMP synthase (cGAS) [22–24]. Other immunity related factors involved in extracellular recognition include so called scavenger receptors [25], which recognize a variety of ligands, and secreted lectins, that specifically bind to the carbohydrate moieties of bacterial cell walls. Particular classes of the latter are also able to initiate the complement system via the lectin-pathway [26]. None of these components have been described for placozoans. This represents a significant gap since deviations from the well investigated immunity mechanisms of vertebrates appear to be quite common among Metazoa. Deviations may include the absence of particular immunity genes or pathways due to the emergence of a lineage before these had evolved, but also lineage specific gene losses or expansion of gene families. The latter mirrors the various phyla’s different evolutionary routes to accomplish recognition and management of harmful or beneficial microorganisms. We therefore examined the *Trichoplax* sp. H2 genome for genes involved in innate immune recognition and downstream signaling. The *Trichoplax adhaerens* reference genome [17] has not been specifically included in our results because both genomes are highly related [20] and the published gene models of the latter were found to be less complete and also inferior with respect to multi-domain genes. Overall, the reference genome did not contribute more information to the presence or absence of immunity related genes.

## Results and discussion

### True toll-like receptors are absent in Placozoa but TLR-like recognition may be organized differently

TLRs are transmembrane (TM) receptors that comprise extracellular leucine-rich-repeats (LRRs), capable of selectively binding foreign ligands like lipoproteins, lipopolysaccharides, peptidoglycans, ssRNA, dsRNA, (unmethylated) CpG-DNA or flagellin, depending on the receptor subtype [21]. Upon binding of the ligand, the cytoplasmic Toll/interleukin-1 receptor (TIR) homology domain associates with the TIR-domain of the adapter protein MyD88, which itself recruits an Interleukin-1 receptor-associated kinase (IRAK) via the association of both of the protein’s Death domains (DDs) [27]. Branches of downstream effectors terminate with the activation of the transcription factors NF-κB, AP-1 or Interferon regulatory factor (IRF), leading to the expression of inflammatory cytokines and other immune genes [21].

To determine if TLR signaling is present in Placozoa, we screened the predicted proteins of the *Trichoplax* sp. H2 genome for TLR pathway orthologs via BLAST and KEGG-, EggNOG- and OrthoMCL-mapping, and further validated putative orthologs by phylogenetic analyses and the presence of the necessary domains using the HMM based domain classification of InterProScan 5 [28]. The latter was also used to identify and characterize TIR-domain containing predictions. In addition, modeling with HHpred [29] was used for certain candidate genes as a more sensitive approach to verify the presence or absence of domains that are otherwise difficult to detect (e.g. because of a higher divergence from the consensus in placozoans).

We found 16 proteins containing a TIR-domain (Additional file 1: Figure S1). Eight of these belong to a class of “evolutionary conserved TIR-domain containing” (ecTIR-DC) proteins that appear to be absent in vertebrates but are widespread among invertebrates and for which a function as adaptors or regulators in immunity-related signaling has been hypothesized [30]. Among these are the only two placozoan TIR-domain proteins that also contain LRRs. Both gene products (classified as ecTIR-DC 14 II in [30]) are 3000 amino acids (AAs) large, lack a TM domain and contain several other domains, making any functional prediction speculative.

While these analyses detected no canonical TLRs in *Trichoplax* sp. H2*,* we further examined if recognition via TLRs may be similarly organized as in *Hydra*, where two separate membrane proteins appear to serve the same function: one containing the extracellular LRRs, the other containing the intracellular MyD88 recruiting TIR-domain [2, 31]. We found nine proteins with extracellular LRRs and a predicted single TM, another potential candidate additionally contained immunoglobulin-like domains (Additional file 1: Table S1). Modeling with HHpred revealed no further intra- or extracellular folds or domains, potentially pointing to a different function, and in two proteins the predicted secondary structure was found to be most similar to that of bilaterian TLRs. For the role of a cytoplasmic transducer we found no TIR-only prediction with a TM. Instead, we found a possible candidate in a SEFIR-domain containing transmembrane protein (Additional file 1: Figure S1). SEFIR-domains are related to TIR-domains and are also thought to be involved in homotypic interactions with other TIR/SEFIR-containing proteins [32]. However, SEFIR-domains are also typical for interleukin-17 (IL-17) receptors which are further characterized by extracellular fibronectin-III-like domains. In the case of the *Trichoplax* SEFIR-domain containing transmembrane protein, neither fibronectin-domains nor other immunoglobulin-like folds were detected by InterProScan and Blast searches did not reveal the slightest resemblance to IL-17 receptors. On the contrary, HHpred detected similarity to IL-17 receptors (and weaker similarity to a TLR) in the region of the SEFIR domain. A reported single hit of a fibronectin-domain to the extracellular part was far beyond significance (see Methods) but homology may nevertheless be considered suggestive, given the presence of the SEFIR domain. The relation of this placozoan gene to other known genes, as well as its function, thus remains difficult to deduce. For the potential role of MyD88 we identified two MyD88-like proteins containing a TIR-domain and the DD-related caspase-recruitment domain (CARD) and another MyD88-like protein containing a SEFIR-domain and a DD (Additional file 1: Figure S1). The latter domain composition resembles both, that of CIKS/CIKSL adaptor proteins involved in IL-17 signaling, and that of MyD88 [33]. HHpred modeling found significant similarity to MyD88 in the region of the DD and to interleukin-17 receptors in the region of the SEFIR domain, which, again, complicates speculations about the protein’s function. However, it is also possible that some of the placozoan proteins that have been found to contain only TIR-domains, or those that belong to the widespread ecTIR-DC proteins, serve as alternative signaling adaptors to MyD88 [21, 30, 34].

We can thus summarize that placozoans possess a variety of TIR/SEFIR-domain containing proteins and putative LRR-receptors that may act in the context of TLR recognition and signaling. A situation resembling that of *Hydra*’s bipartite TLRs. However, although *Hydra*’s atypical TLRs are also supported by functional assays [31], we have to note that their proposed TIR-domain containing transmembrane proteins (HyTRR-1, HyTRR-2) have been found to likely adopt an extracellular immunoglobulin-like fold and thus seem rather related to interleukin-1 receptors [30]. If this contradicts a role in TLR recognition or simply emphasizes a derived status of hydrozoans within Cnidaria (anthozoans possess true TLRs [2]), has yet to be resolved. In the case of placozoans, the recognition arm of TLR signaling remains speculative since true TLR orthologs are absent and functional data are missing. It is also possible that other, yet unknown, receptors provide the input for TLR signaling.

### The presence of TLR pathway orthologs indicates primordial TLR signaling

**Fig. 1:**
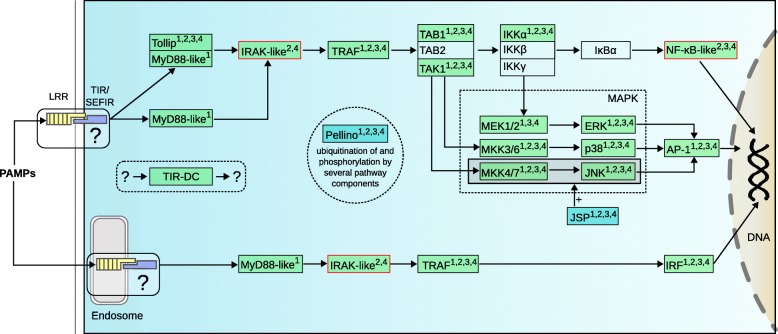
Simplified reference TLR signaling pathway projected onto putative homologs in *Trichoplax* sp. H2. True Toll-like receptors are absent but many components of the downstream signaling cascade are present. Support for the presence and homologous function of pathway components is indicated by filled boxes and superscript letters, where 1: is supported by domain composition, 2: is supported by KEGG annotation, 3: is supported by two of either EggNOG OG, OrthoMCL OG or best Swiss-Prot hit, 4: is supported by phylogenetic validation. Uncertainty regarding homology because of missing domains is indicated by a red outline. For example, *Trichoplax* possesses several IRAK-like genes but all are lacking the DD of true IRAK genes. In the case of the identified NF-κB-like protein, an RHD and ANK repeats are present but a DD is absent. Question marks denote unknown down- or upstream signaling components: Since true Toll-like receptors are absent and a bipartite receptor (extracellular LRR, intracellular TIR/SEFIR) is hypothetical, the recognition part in TLR signaling remains speculative. Similarly, some of the identified TIR domain containing (TIR-DC) proteins may function in TLR signaling as alternative adaptor molecules, but their upstream input as well as their targets have yet to be identified. The function of the missing IκBα ortholog may be fulfilled by other placozoan ANK containing proteins. The MyD88-independent pathway has been omitted since it is assumed to have emerged in chordates. See also Additional files 1, 2 and 3

We identified many orthologs of the downstream effectors of the canonical TLR pathway (Fig. 1; Additional file 1: Table S2; Additional file 2: Dataset S1; Additional file 3: Dataset S2), indicating the presence of components for primordial TLR signaling in Placozoa. There are, however, important exceptions: While the pathway leading to AP-1 via mitogen-activated protein kinases (MAPK) seems complete, the path to NF-κB lacks the NF-kappa-B inhibitor alpha (IκBα), which, after phosphorylation by inhibitor of nuclear factor kappa-B kinases (IKKs), enables translocation of NF-κB to the nucleus (reviewed in [35]). However, the only conserved domains present in IκBα and related proteins are ankyrin-repeats (ANK) and the detection of putative placozoan homologs may thus be impeded if they deviated substantially from the bilaterian consensus. It is also possible that an unrelated protein among the roughly 200 ANK containing proteins in *Trichoplax* sp. H2 fulfills this function. Furthermore, the here identified placozoan NF-κB-like protein (p100-subunit-like) deserves some attention. While InterProScan identified only ANK repeats, the analysis with HHpred supports complete Rel homology and Rel homology dimerisation domains (RHD, IPT). Interestingly, two adjacent predictions in the genome contain well recognizable RHDs only, which adds up to three clustered members of the NF-κB/Rel family in placozoans, but otherwise these two appear to have no relation to known members of this gene family. The lack of a DD in the NF-κB- p100-like protein further indicates that the function and regulation of NF-κB complexes in Placozoa deviates from canonical pathways.

Since sponges and Cnidaria do possess complete NF-κB (in current NCBI databases, cf. [36, 37]), the question if placozoans have a disrupted NF-κB or represent a stage before acquisition of the DD depends on the actual phylogeny at the base of the Metazoa, which at this point in time is controversial (e.g. see [38]). However, if Placozoa are basal in the phylogeny, then the evolutionary scenario would be that they represent the stage before acquisition of the DD. If on the other hand, Placozoa were intermediate to sponges and Cnidaria, then the inference would be a disrupted NF-κB.

The absence of TRIF and TBK1 orthologs further indicates that Placozoa lack the MyD88-independent pathway, consistent with the hypothesis of its emergence in chordates [39], but the most important deviation from the reference pathway is the unclear orthology of complete IRAK family genes. While we found several IRAK-related kinases via BLAST, KEGG- or EggNOG-mapping, all of them lack a DD which is thought to be responsible for association with MyD88 [27]. Many of these IRAK related placozoan protein kinases lie in a tight genomic cluster and thus seem to be placozoan specific paralogs produced by gene duplication. However, while Porifera possess reasonable IRAK orthologs with a DD (e.g. *A. queenslandica*: XP_011406220.2) this seems not to be the case for Cnidaria (c.f. [37, 40]) where all IRAK-like genes lack a DD, at least the closest cnidarian hits for bilaterian IRAKs in current databases such as UniProt, NCBI or Compagen. Nevertheless, it is generally assumed that TLR signaling is present in Cnidaria and functional analyses in *Hydra* have shown that several downstream targets of TLR signaling are affected after silencing of MyD88 [41], indicating that the pathway is functional even in the absence of a clear IRAK ortholog and the same could be true for placozoans. In the absence of IRAK, other kinases containing a DD could serve similar functions. In the predicted gene models of *Trichoplax* sp. H2 we were, however, only able to identify one kinase containing a Death-like domain (DLD).

### *Trichoplax* sp. H2 contains a rich and unique scavenger receptor repertoire

Scavenger receptors (SR) are another class of cell surface receptors whose functional diversity has only recently been recognized. They are able to bind a large variety of ligands and their primary role seems to be the clearance from unwanted compounds like cellular debris, pathogens or other foreign particles by endocytosis [25]. SRs constitute a structurally heterogeneous group, but most receptors associated with scavenger receptor activity are characterized by the presence of either C-type lectin domains (CTLD), scavenger receptor cysteine-rich domains (SRCR) or the CD36 domain. Generally, the SR repertoire seems to be greater in invertebrates, likely because of the absence of an adaptive immune system [3, 42, 43]. Their capacity to manage internalization of extracellular ligands by endocytosis makes them suitable candidates not only for detecting and fending off invasive pathogens but also to mediate symbiosis with intracellular bacteria.

**Fig. 2:**
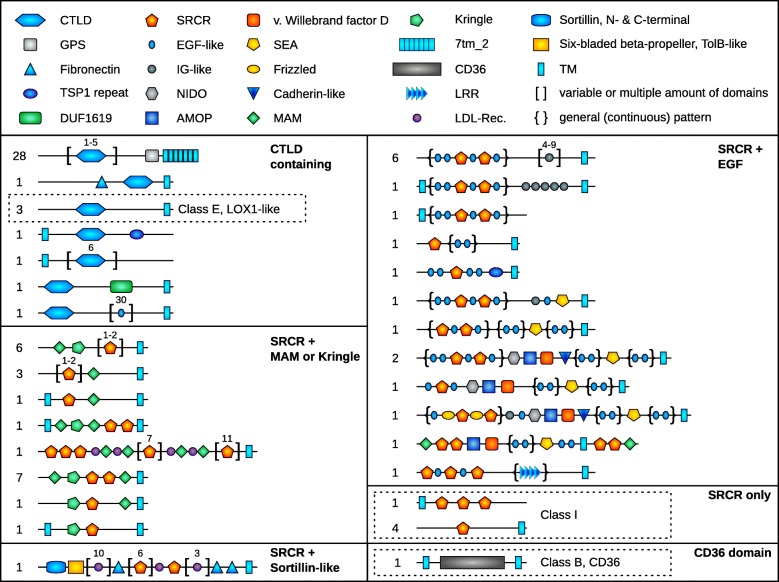
The scavenger receptor repertoire in *Trichoplax* sp. H2 shows a remarkable domain diversity but only mammalian classes B, E and I are recognizable. The combination of CTLDs with GPCRs of the secretin/adhesion family is unique. Numbers on the left represent the count of predicted proteins with the respective domain pattern. Numbers above brackets indicate the range or amount of the same domain. An additional glycoprotein hormone receptor containing one CTLD has been omitted. See also Additional file 4: Dataset S3A

We found a large repertoire of transmembrane proteins containing SRCR domains or CTLDs: Together with a single CD36 ortholog, the *Trichoplax* sp. H2 genome harbors 82 putative scavenger receptors, 45 containing SRCR domains, 36 CTLDs (Fig. 2; Additional file 4: Dataset S3A). Like in Cnidaria [43], no overlap between SRCR domain and CTLD domain containing proteins was observed. As expected for invertebrates, the number of scavenger receptors containing the above domains exceeds by far that of mammals (e.g. 11 in human) and also that of most Cnidaria so far investigated (12–38), with the exception of *Aiptasia pallida* (72) where it is more or less the same [43]. However, the diversity of domain composition in placozoan SRs is greater than in the latter two animal groups. While the receptors containing either SRCR, CTLD or CD36 are composed of 6 different domains in mammals and 13 in Cnidaria [25, 43], more than 20 different domains are present in placozoan SRs and most combinations appear restricted to the phylum. Accordingly, most scavenger receptors found in *Trichoplax* sp. H2 cannot be assigned to well recognized mammalian classes, with the exception of Class B, E and I.

### The majority of CTLD containing scavenger receptors in placozoans are G protein–coupled receptors

The SR complement of placozoans also differs in another unique aspect as 28 of 36 CTLD containing receptors bear seven transmembrane helices instead of a single transmembrane domain and are G protein–coupled receptors (GPCRs) of the Secretin/Adhesion family. To our knowledge these are the only described GPCRs whose extracellular domains consist of CTLDs. The one exception is *Branchiostoma* which harbors one adhesion GPCR containing an N-terminal CTLD, albeit together with other extracellular domains [44]. This suggests that CTLD mediated recognition of molecular patterns in Placozoa is linked to GPCR signaling, trafficking and endocytosis [45]. Furthermore, β-arrestin mediated endocytosis also couples GPCRs to the MAPK pathway. Hence, binding and internalization of targets could trigger a variety of cellular events, including defense or management of symbiotic microorganisms. In this context it is noteworthy that we found a large repertoire of arrestins (defined by the presence of N- and C-terminal Arrestin-like domains) in the *Trichoplax* sp. H2 genome (Additional file 4: Dataset S3B): in addition to one ortholog of β-arrestin, twenty three α-arrestins, related to the mammalian genes ARRDC2–4, are present, seventeen of which reside in a tight cluster on scaffold 76. Compared to mammals (two β-arrestins, two visual arrestins, six α-arrestins [46]), *Nematostella* (UniProt: one β-arrestin, three α-arrestins) and *Amphimedon* (UniProt: one β-arrestin, six α-arrestins) this gene number constitutes a unique expansion of the arrestin family in Placozoa. Even though the role of α-arrestins in GPCR signaling is less well understood, they are also known to interact with GPCRs in concert with β-arrestin [46]. This observation indicates that GPCR mediated endocytosis in placozoans plays an important role for defense in particular and GPCR signaling in general.

### Secreted proteins containing fibrinogen-related domains appear to play an important role in extracellular defense

Before pathogens come into contact with cell-surface receptors, the line of first defense consists of secreted lectin-like proteins that are able to recognize carbohydrates associated with bacterial cell walls and either agglutinate pathogens, or coat and opsonise them to enhance phagocytosis. Possible candidates, among others, are secreted proteins that contain CTLDs [47] or fibrinogen-related domains (FReDs) [48]. Twelve predictions without a TM were identified harboring 1–3 CTLDs, eight of which contain a signal peptide (SP) and are thus likely to be secreted factors (Additional file 4: Dataset S3C). Fibrinogen-related domains on the other hand were previously claimed to be absent in placozoans [48]. However, using the InterProScan annotation we were able to detect 18 FReD containing gene models with SUPERFAMILY member SSF56496 (Fibrinogen, C-terminal globular domain; Additional file 4: Dataset S3C). While 2 of these are large proteins related to fibrillin, the remainder are around 250 AA in length, bear a single FReD and an SP (except one) and are mostly organized in three clusters in the genome. The PANTHER classification system [49] classifies them as intelectins, a novel type of soluble lectins (X-type lectin), which seem remotely related to ficolins and have been shown to selectively recognize bacterial glycans [50]. Moreover, an additional 15 gene models (11 with SP) are classified as intelectins by PANTHER and all of these, except 2, are genomic neighbors of the above, producing 5 clusters of 2–9 genes each. In 28 of these 31 genes, modeling with HHpred further supports the presence of a FReD (the remaining 3 predictions are probably truncated). This observation indicates that most of the putative intelectins belong to the same gene family but contain FReDs that are difficult to detect as a result of their phylogenetic distance to related sequences, which are the basis of canonical domain models. This could also be the reason why the identified FReDs in placozoan intelectins are comparatively short compared to vertebrate intelectins (up to 70 AAs versus 200 AAs).

Vertebrate and placozoan intelectins thus only align well in the region of the shorter placozoan FReD (restricted to the N-terminal portion of placozoan intelectins) while the latter also show high sequence similarity in the C-terminal portion (Additional file 5: Figure S2; Additional file 6: Dataset S4). Most placozoan sequences share 10 conserved cysteine residues (4–5 with vertebrate intelectins; complete alignment in Additional file 6: Dataset S4), suggesting that they are essential to form either intrachain or interchain disulfide bonds. The latter could serve to form intelectin oligomers, as is the case in human Intelectin-1 [50], which probably increases agglutination of trapped bacteria. While mammals possess up to 6 intelectins, teleosts have 9 and tunicates 22 intelectins [51], there are up to 31 intelectins in *Trichoplax* sp. H2. The apparent expansion of this gene family in placozoans suggests that intelectins play an important role in extracellular defense of the animal.

Some lectins, like the FReD containing ficolins and the CTLD containing mannose-binding protein (MBP), are known to initiate the lectin-complement-pathway [26] which represents an immediate response system for recognition and defense. In the cnidarian *Aiptasia* it has also been shown that the complement system is involved in the management of the cnidarian-dinoflagellate symbiosis [52]. It is thus tempting to speculate if the placozoan intelectins or secreted CTLD proteins could serve a similar function. However, intelectins, as well as the placozoan secreted CTLD proteins, lack collagen repeats or coiled-coils that could mediate oligomerization similar to ficolins or MBP, which is needed to trigger the lectin-pathway. Moreover, placozoans apparently lack a complement system at all since most of its factors are absent. For example, neither KEGG pathway mapping nor screening for the necessary domains revealed genes for the key factors Mannan-binding lectin serine protease (MASP) or the complement factors B, C2, C3 or C4. With respect to the factors C3/C4, the only members of the Alpha-2-macroglobulin family present in *Trichoplax* sp. H2 are two CD109 genes which are not involved in the complement system. We only identified a single C1q-like factor (Additional file 4: Dataset S3C) bearing a clear C1q domain, and collagen repeats that are only recognizable by structural homology prediction with HHpred. In vertebrates, C1q plays an important function in the classical complement pathway [26]. In invertebrates, which lack adaptive immunity, C1q factors are assumed to rather act as lectins in immune recognition and have undergone expansion in several taxa [53]. The latter, apparently, does not apply to Placozoa.

### Intracellular defense in Placozoa does not conform to the classical NOD-like receptor pathway and RIG-I-like receptors or cGAS-STING signaling are absent

NLRs mediate recognition once pathogens or their components have entered the cytoplasm [22, 54]. These proteins contain a central NOD domain (NACHT or NB-ARC) for nucleotide binding and self-oligomerization and C-terminal LRRs for molecular pattern recognition. An N-terminal effector domain mediates protein-protein interactions with downstream targets. Effector domains are members of the Death clan like PYD, CARD or the Death Effector Domain (DED). In the canonical NOD pathway, the receptors oligomerize after binding of PAMPs and expose their CARDs, enabling interaction with the CARD containing serine-threonine kinase RIPK2 which activates NF-κB and MAPK signaling [22]. Other NLRs contain tetratricopeptide (TPR), WD40 or ANK repeats instead of LRRs and seem not involved in PAMP recognition (e.g. [54, 55]). RIG-I-like receptors detect viral RNA and are composed of a central DEAD/DEAH box helicase domain, an N-terminal CARD and a C-terminal regulatory domain [23]. A further mechanism for detecting foreign cytosolic DNA is the cGAS-STING pathway [24].

**Fig. 3:**
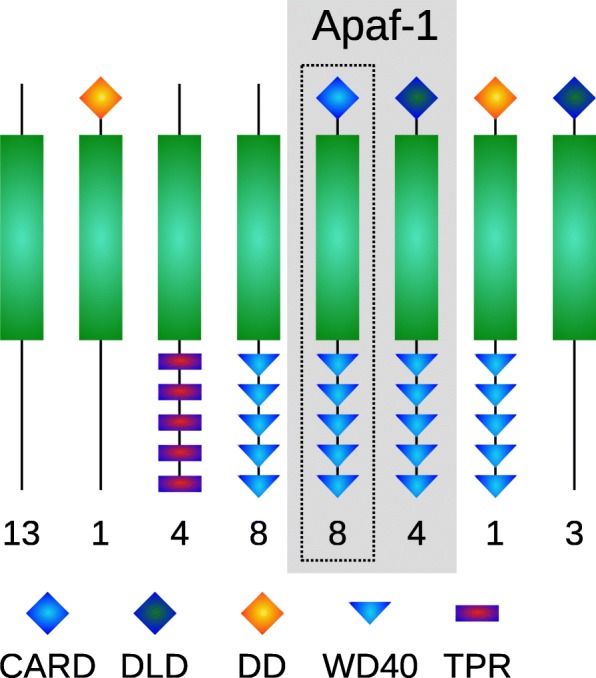
Domain composition of the NOD-like receptor repertoire in *Trichoplax* sp. H2. The central NOD-domain (NACHT/NB-ARC) is depicted in green. Possible Apaf-1 homologs are shaded in grey, clear homologs are bordered by a dotted line. Numbers represent the count of predicted proteins with the respective domain pattern. See also Additional file 4: Dataset S3D

While genes for cGAS, STING or RIG-I-like receptors were not found in *Trichoplax* sp. H2, 42 predicted genes containing a central NACHT/NB-ARC-domain (NACHT 3, NB-ARC 39) were identified (Fig. 3; Additional file 4: Dataset S3D). None of these contain LRRs and are thus unlikely to be involved in recognition of invading pathogens. In addition, no RIPK1/2 ortholog could be found in the *Trichoplax* sp. H2 genome, which is similar to the situation in cnidarians [55, 56]. The C-terminal repeats of placozoan NLRs are always either WD40 or TPR type repeats. Effector domains are predominantly CARDs or Death-like domains (DLDs) and in two cases DDs. These domain combinations of placozoan NLRs mostly resembles that of *Hydra*, which also does not contain any NOD-like receptors with LRRs, in contrast to anthozoans [54, 55].

### Apaf-1 and controlled suicide as a means of defense

The NOD-like repertoire of placozoans also differs remarkably in another aspect to bilaterians and cnidarians: while most animals possess only one homolog of the Apoptotic protease activating factor-1 (Apaf-1), placozoans harbor several paralogs in their genome. If we consider DLDs as difficult to detect putative CARDs, *Trichoplax* sp. H2 harbors twelve Apaf-1 homologs, possibly even more because several Apaf-1 genes reside in clusters of NB-ARC containing genes but some gene models are missing either WD40 repeats or a CARD/DLD (Fig. 3; Additional file 4: Dataset S3D).

Apaf-1 plays a key role for the initiation of the mitochondrial pathway of apoptosis [57]. It forms an oligomeric apoptosome and ignites the action of downstream caspases. The trigger is the released danger signal cytochrome c resulting from mitochondrial damage, a possible result of an infection. Apaf-1 thus enables controlled suicide of impaired cells. The death signal is passed from Apaf-1 to Caspase-9 via the association of both of the protein’s CARDs. Although H2 seems not to possess a clear Caspase-9 ortholog, it harbors two neighboring genes containing a caspase-like domain and an N-terminal CARD (Additional file 4: Dataset S3D), suggesting a similar function in apoptosis.

The presence of many Apaf-1 paralogs in placozoans suggests that controlled cell death after damage (e.g. by infection) plays a fundamental role in maintaining somatic and genetic integrity of the animal. Rather than triggering inflammatory responses, the cell is sacrificed once pathogens have escaped defense mechanisms and captured the cell. There are two reasons this strategy would make sense. First, the remarkable regenerative capacity of placozoans would be a contributing factor to how this hypothetical mechanism works and second, placozoans do not possess any organs whose accurate function has to be secured at all costs. Even the sacrifice of the entire animal seems plausible, if we assume that a local population mainly reproduces asexually and consists of genetically identical individuals. The selective advantage for a local genotype could thus lie in a rapid suicide of single infected animals to prevent spreading a disease to the entire population.

## Conclusions

The immunity related pathways and genes found in the placozoan 16S haplotype H2 show several deviations from the common features of innate immune systems that have been characterized from phylogenetic branches of Bilateria to date. Due to the inconsistencies in related gene repertoires among other basal Metazoa (Porifera, Cnidaria) it is not always clear if these deviations (e.g. unclear orthology of IRAK and incomplete NF-κB) are the result of gene or domain loss, or because Placozoa simply predate the origin of such genes. Most remarkable is the presence of many genes for downstream TLR signaling while true Toll-like receptors are absent. Although we found candidate genes that may play a role in TLR recognition, this has to be proven experimentally. It is also possible that yet unknown receptors provide the input for TLR signaling in Placozoa. Other identified genes clearly constitute placozoan specific expansions of their immune repertoire (e.g. scavenger receptors, intelectins, Apaf-1). The distinct gene repertoire of placozoans further indicates that different metazoan phyla have embarked on different evolutionary routes to accomplish recognition of non-self in particular fields, but in general they use similar or the same core mechanisms. In any case, the complexity and amount of the found immunity genes suggests that Placozoa have sophisticated means to protect themselves against microbial invasion and to manage and maintain their associated microbiota - though experimental data is still needed for verification and to understand how they utilize this repertoire.

## Methods

### Functional annotation of *Trichoplax* sp. H2 predicted genes

The sequencing and annotation of the *Trichoplax* sp. H2 genome has been described in Kamm et al. (2018) [20]. Briefly, gene prediction was carried out using the evidence based Maker pipeline [58] and functional annotation of the predicted proteins was carried out using InterProScan 5 (5.19–58.0) [28] with the following analyses: CDD-3.14, SignalP_EUK-4.1, PIRSF-3.01, Pfam-29.0, SignalP_GRAM_POSITIVE-4.1, TMHMM-2.0c, PRINTS-42.0, ProSiteProfiles-20.119, PANTHER-10.0, Coils-2.2.1, Hamap-201,605.11, ProSitePatterns-20.119, SUPERFAMILY-1.75, ProDom-2006.1, SMART-7.1, SignalP_GRAM_NEGATIVE-4.1, Gene3D-3.5.0 and TIGRFAM-15.0. Signal peptide (SP) and transmembrane (TM) predictions for innate immunity related genes were also carried out using the online server of Phobius [59]. Annotation of predicted proteins also included BLASTP [60] searches against Swiss-Prot (cutoff e-value 1e-5) and KEGG pathway mapping using KAAS [61]. Assignment to orthologous groups was done using the online servers of OrthoMCL (http://orthomcl.org/orthomcl/proteomeUpload.do [62]) and eggNOG-mapper (http://eggnogdb.embl.de/#/app/emapper [63]).

### Identification of innate immunity related genes in *Trichoplax* sp. H2

For the identification and description of innate immunity genes in placozoans we preferentially used the predictions of the *Trichoplax* sp. H2 genome [20]. Gene models of the *Trichoplax adhaerens* reference genome [17] were not specifically included because 1. both genomes are highly related and are expected to contain more or less the same genes, 2. the reference genome is less complete and 3. the corresponding predictions are less complete, especially regarding multi-domain genes. If orthologs of important immunity related genes could not be identified in *Trichoplax* sp. H2, the reference genome and the respective transcriptomes (see Kamm et al. 2018 [20]) of the two species were screened for their presence. Overall, neither the *Trichoplax adhaerens* reference genome nor the transcriptomes provided additional information.

Candidate orthologous genes of the Toll-like receptor (TLR) pathway were identified via KEGG mapping (see Kamm et al. 2018 [20]), best BLASTP hits of the predicted proteins against Swiss-Prot and by blasting respective metazoan orthologs against the predicted proteins. The candidate genes were further verified by mapping the predicted proteins to orthologous groups via OrthoMCL and EggNOG and by the presence of all necessary domains using the output of the InterProScan 5 functional annotation. The InterProScan results were also used to identify immunity and molecular pattern recognition related gene products based on their domain composition. Thus, in the functional annotation we looked for gene products containing (I) leucine-rich repeats (LRRs: IPR032675), the Toll-Interleukin receptor homology (TIR: IPR000157) or SEFIR (IPR013568) domain and could fulfill Toll-like receptor function; (II) the C-type lectin domain (CTLD: IPR001304), the scavenger receptor cysteine-rich (SRCR: IPR001190) domain or the CD36 (IPR002159) domain of putative scavenger receptors; (III) fibrinogen-related domains (FReDs: IPR002181); (IV) NACHT (IPR007111) or NB-ARC (IPR002182) domains of NOD-like receptors; (V) the C-terminal domain of retinoic acid inducible gene I (RIG-I: IPR021673) and (VI) the Mab-21 domain (IPR024810) of cyclic GMP-AMP synthase or IPR029158/IPR033952 of the stimulator of interferon genes protein for the presence of cGAS-STING signaling. Key complement factors were searched for via KEGG mapping and by screening the H2 proteins’ InterProScan annotation for the necessary domains (as defined by the InterProScan annotation of *Homo sapiens* orthologs in Swiss-Prot). For certain candidate immunity genes, modeling with HHpred [29], implemented in the MPI Bioinformatics Toolkit [64], was used as a more sensitive approach to ensure the presence or absence of domains that are otherwise difficult to detect because of a potential higher divergence from the consensus in placozoans. HHpred was run with default parameters and hits with a probability ≥90% and an E-value ≤0.1 were considered significant. Signal peptide and transmembrane region predictions were taken from the InterProScan 5 annotation (SignalP_EUK-4.1 [65], TMHMM-2.0c [66]) and also inferred using the Phobius web server [59].

### Orthology validation of TLR pathway genes using phylogenetic analyses

The putative placozoan orthologs of the TLR pathway (Table S2) were subjected to phylogenetic analysis with potential orthologs from their respective gene family members. The potential gene family member sequences were obtained using a modification of the method outlined in Rosenfeld et al., 2016 [67]. Briefly, a cDNA collection of complete and annotated genomes from NCBI was compiled using the taxa (and links) in Additional file 7: Dataset S5. These cDNA sequences were then processed with Transdecoder [68] to produce the best ORFs for each gene and collected into a single BLAST database. Because the human cDNAs contain the most reliable annotation, these ORFs were then queried against the full database of animal ORFs using TBLASTX to identify matches (cutoff value 1e-10). The most representative ORF for a gene in a particular taxon was determined by taking the BLAST hit with the lowest eValue. The matches for each of the genes per taxon were collected so that we had individual files with all of the orthologs of a particular human gene. Each of the TLR pathway genes of H2 was then used as a query for this database to give us a collection of putative orthologs for the H2 gene. This procedure resulted in orthologous groups with unique entries for a maximum number of animal taxa.

Phylogenetic matrices were built by combining the orthologous groups into a single matrix and by aligning the amino acid sequences with MAFFT (default settings). Poorly aligned regions in these alignments were then masked using *Homo sapiens* sequences as a guide. Phylogenetic analysis was accomplished using parsimony in TNT [69]. We chose this method because it inherently relies on diagnostic changes to place terminals in a phylogenetic context [70]. The TNT analyses used 1000 ratchet replicates saving 1000 trees at each replicate and consensus trees were used to summarize the phylogenetic searches. For a group of orthologs that make up a gene family (e.g. such as MEP1, TRAF1, TRAF2, TRAF3, TRAF4, TRAF6 and TRAF7) we combined the genes for these gene family members into a single matrix and analyzed them as a gene family unit.

## Additional files

Additional file 1: **Figure S1.** TIR/SEFIR-domain containing proteins in *Trichoplax* sp. H2. **Table S1.** Possible candidates for the extracellular part of a hypothetical bipartite TLR-like receptor in *Trichoplax* sp. H2. **Table S2.** The TLR pathway related gene repertoire in *Trichoplax* sp. H2 apart from TIR-domain containing genes. (PDF 83 kb)

Additional file 2: **Dataset S1.** Trees of the phylogenetic validation of the TLR pathway genes. (PDF 1122 kb)

Additional file 3: **Dataset S2.** Conserved domain alignments of the TLR pathway genes. (PDF 2446 kb)

Additional file 4: **Dataset S3.** Spreadsheet containing the protein domain summary and the locus_tags of: **A** The *Trichoplax* sp. H2 scavenger receptors **B** The *Trichoplax* sp. H2 arrestins **C** The *Trichoplax* sp. H2 lectin-like secreted proteins (CTLD, FReD or C1q containing) **D** The *Trichoplax* sp. H2 NOD-like receptors. (XLSX 32 kb)

Additional file 5: **Figure S2.** Vertebrate and placozoan intelectins aligned in the region of the fibrinogen-related domain (FReD) of placozoan intelectins. (PDF 117 kb)

Additional file 6: **Dataset S4.** Complete alignment of the vertebrate and placozoan intelectins shown in Additional file 5: Figure S2 (Fasta format). (FA 15 kb)

Additional file 7: **Dataset S5.** Spreadsheet with the list of taxa used for phylogenetic validation of the TLR pathway genes. Links are given for the NCBI download source of the respective cDNA databases. (XLSX 18 kb)

## Abbreviations

AA: Amino acid
ANK: Ankyrin-repeats
Apaf-1: Apoptotic protease activating factor-1
CARD: Caspase-recruitment domain
cGAS: Cyclic GMP-AMP synthase
CTLD: C-type lectin domain
DD: Death domain
DED: Death Effector Domain
DLD: Death-like domain
ecTIR-DC: Evolutionary conserved TIR-domain containing
FReD: Fibrinogen-related domain
GPCR: G protein-coupled receptor
IKK: Inhibitor of nuclear factor kappa-B kinase
IL-17: Interleukin-17
IPT: Rel homology dimerisation domain
IRAK: Interleukin-1 receptor-associated kinase
IRF: Interferon regulatory factor
IκBα: NF-kappa-B inhibitor alpha
LRR: Leucine-rich-repeat
MAPK: Mitogen-activated protein kinase
MASP: Mannan-binding lectin serine protease
MBP: Mannose-binding protein
MyD88: Myeloid differentiation primary response 88
NF-κB: Nuclear factor kappa-B
NLR: NOD-like receptor
NOD: Nucleotide binding and oligomerization domain
PAMP: Pathogen-associated molecular pattern
PRR: Pattern recognition receptor
PYD: Pyrin domain
RHD: Rel homology domain
RIG-I-like: Retinoic-acid inducible gene I like receptor
SP: Signal peptide
SR: Scavenger receptor
SRCR: Scavenger receptor cysteine-rich domain
STING: Stimulator of interferon genes receptor
TIR: Toll/interleukin-1 receptor homology domain
TIR-DC: TIR-domain containing
TLR: Toll-like receptor
TM: Transmembrane
TPR: Tetratricopeptide
