# Supplementary material for: Innate immunity in the simplest animals – placozoans
Source: BMC Genomics. 2019 Jan 5;20:5. doi: 10.1186/s12864-018-5377-3 (PMC6321704; doi:10.1186/s12864-018-5377-3)
Supplement: Supplementary file 1 — Figure S1. TIR/SEFIR-domain containing proteins in Trichoplax sp. H2. Table S1. Possible candidates for the extracellular part of a hypothetical bipartite TLR-like receptor in Trichoplax sp. H2. Table S2. The TLR pathway related gene repertoire in Trichoplax sp. H2 apart from TIR-domain containing genes. (PDF 83 kb) [file 12864_2018_5377_MOESM1_ESM.pdf]

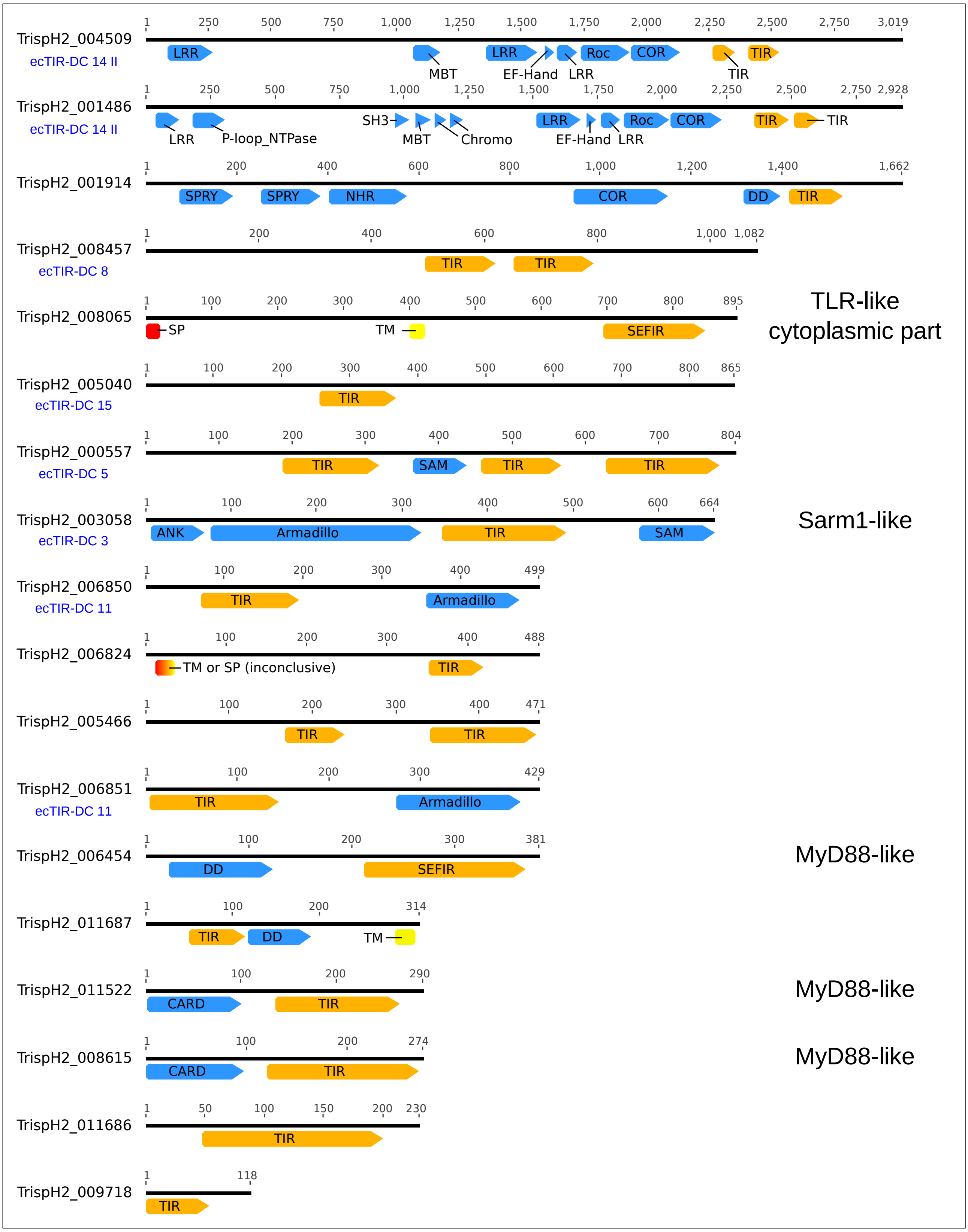

Figure S1 TIR/SEFIR-domain containing proteins in *Trichoplax* sp. H2. True Toll-like receptors are absent. The only proteins that contain a TIR-domain and LRRs are two large proteins lacking a transmembrane domain and harboring several additional domains. Hypothetical, similar to *Hydra*, the function of the intracellular part of a TLR-receptor could be fulfilled by a transmembrane protein with a cytoplasmic TIR/SEFIR-domain that is

capable of homotypic interactions with other such domains. A possible candidate is a protein predicted to contain an SP, a TM and a cytoplasmic SEFIR-domain. The role of the intracellular adaptor (MyD88) could be fulfilled by MyD88-like proteins. Other TIR-domain containing proteins may also serve as alternative adaptors or regulators in TLR signaling. Indicated are the locus_tags of the *Trichoplax* sp. H2 proteins. Proteins that belong to the group of the recently proposed “evolutionary conserved TIR-domain containing” (ecTIR-DC) proteins [1] are indicated by the respective family name below their locus_tag. Shown are only IPR domains identified by InterProScan. Proteins are not drawn to scale but sorted into size classes. Domains are drawn to scale within each protein.

Table S1 Possible candidates for the extracellular part of a hypothetical bipartite TLR-like receptor in *Trichoplax* sp. H2. These predicted proteins only contain leucine-rich-repeats (LRR) for ligand-binding and a transmembrane region. A further candidate additionally contains immunoglobulin-like domains. Analyses using HHpred revealed no further intra- or extracellular folds and that candidate genes TrispH2_006375 and TrispH2_011696 are most similar to bilaterian TLRs. The genes TrispH2_009860-62 are organized in tandem in the genome.

locus_tag	Size (AA)	SignalP/TMHMM prediction	Phobius prediction	Prediction of LRR location (Phobius)	other domains
TrispH2_000076	976	SP+TM	SP+TM	non-cytoplasmic	IG-like
TrispH2_000329	611		TM	non-cytoplasmic	
TrispH2_006375	650	SP+TM	SP+TM	non-cytoplasmic	
TrispH2_009003	884	TM	TM	non-cytoplasmic	
TrispH2_009860	478	SP	SP+TM	non-cytoplasmic	
TrispH2_009861	488	SP	SP+TM	non-cytoplasmic	
TrispH2_009862	543	SP	SP+TM	non-cytoplasmic	
TrispH2_011004	295	TM	TM	non-cytoplasmic	
TrispH2_011248	545	SP	SP+TM	non-cytoplasmic	
TrispH2_011696	403		TM	non-cytoplasmic	

Table S2 The TLR pathway related gene repertoire in *Trichoplax* sp. H2 apart from TIR-domain containing genes. Shown are BLAST, orthologous mapping and InterProScan results of the candidate genes. Placozoans clearly harbor many genes of the canonical pathway. Ambiguous orthologs are those of IRAK and NF-κB: Several IRAK related kinases could be found by BLAST and KEGG- or EggNOG-mapping but all of these lack a Death domain (DD). The identified NF-κB related gene similarly lacks a DD. Its Rel homology domain (RHD+IPT) deviates from the consensus such that it could only be identified through structural homology prediction with HHpred.

Gene	locus_tag	Swiss-Prot best hit	KEGG KO mapping	EggNOG mapping	OrthoMCL mapping	IPR domains	IPR family prediction	Phylogenetic assignment	remark
**AP-1 (JUN)**	TrispH2_000596	JUN/AP-1	K04448, JUN	JUN	OG5_131442, JUN	JNK, bZIP	Leuzip_Jun	JUN	
**ERK1/2 (MAPK1/3)**	TrispH2_001959	MAPK1	K04371, MAPK1/3	MAPK3	OG5_126781, MAPK1/3	Protein kinase	MAPK_ERK1/2	MAPK1/3	
**IKK**	TrispH2_007459	CHUK	K04467, IKBKA	IKBKB	OG5_131581, IKBKB	Protein kinase	/	CHUK	
**IRAK**	TrispH2_001168	IRAK2	/	ENOG410ZCW6	OG5_142204	Tyrosine kinase	/	IRAK3	Death domain (DD) is absent in all IRAK related kinases. The kinases with the locus_tags TrispH2_ 009235-009242 reside in a tight cluster of several more protein kinases.
	TrispH2_008689	At1g01540	K04733, IRAK4	PELLE (IRAK4)	OG5_135302	Tyrosine kinase	/	IRAK3	
	TrispH2_009076	IRAK4	/	KOG1187	OG5_142204	Tyrosine kinase	/	IRAK3	
	TrispH2_009235	SD16	/	IRAK1	OG5_135302	Protein kinase	/	IRAK3	
	TrispH2_009237	CRCK2	/	IRAK1	OG5_135302	Protein kinase	/	IRAK3	
	TrispH2_009239	EMS1	/	IRAK1	OG5_135302	Protein kinase	/	IRAK3	
	TrispH2_009240	IRAK4	/	KOG1187	OG5_142204	Protein kinase	/	IRAK3	
	TrispH2_009241	IRAK4	/	KOG1187	OG5_142204	Protein kinase	/	IRAK3	
	TrispH2_009242	MSP1	/	IRAK1	OG5_135302	Protein kinase	/	IRAK3	
	TrispH2_010672	IRAK4	/	PELLE (IRAK4)	/	Tyrosine kinase	/	IRAK3	
**IRF**	TrispH2_009842	IRF2	K10153, IRF2	IRF2	OG5_137622, IRF2	IRF, ZU5, DD	/	IRF2	
	TrispH2_001644	IRF1	/	IRF4	OG5_137622, IRF2	IRF	/	IRF2	
	TrispH2_003387	IRF2	/	IRF4	OG5_137622, IRF2	IRF	/	IRF2	
**JNK (MAPK8/9/10)**	TrispH2_007604	MAPK10	K04440, JNK	MAPK10	OG5_129677, JNK	Protein kinase	MAPK_JNK	MAPK10	
**JSP (DUSP22)**	TrispH2_000711	DUSP22A	K14165, DUSP	DUSP22	/	DSP	DUSP	DUSP22/15	
	TrispH2_000712	DUSP22	K14165, DUSP	DUSP22	OG5_131563, DUSP	DSP	DUSP	DUSP22/15	

**MEK1/2 (MAP2K1/2)**	TrispH2_001322	MAP2K1	/	MAP2K1	OG5_127304, MEK1/2	Protein kinase	/	MAP2K1	
**MKK3/6 (MAP2K3/6)**	TrispH2_004233	MAP2K6	K04432, MKK3	MAP2K6	OG5_131939, MKK3/6	Protein kinase	/	MAP2K3/6	
**MKK4/7 (MAP2K4/7)**	TrispH2_003417	MAP2K4	K04430, MKK4	ENOG410V5YT, MAP2K	OG5_133213, MKK4	Protein kinase	/	MAP2K4	
	TrispH2_003961	MAP2K7	K04431, MKK7	MAP2K7	OG5_133887, MAP2K7	Protein kinase	/	MAP2K7	
**NF-κB**	TrispH2_003408	NFKB2	K02580, NFKB1	NFKB2	OG5_142327, IKBE	ANK repeats	/	NF-κB	Derived RHD and IPT domains identified with HHpred. Two further RHD containing genes 3' in the genome (TrispH2_003409, TrispH2_003410)
**p38 (MAPK11/13)**	TrispH2_004732	MAPK11	K04441, p38	MAPK13	OG5_128610, p38	Protein kinase	MAPK_p38	MAPK11	
**Pellino**	TrispH2_002428	PELI2	K11964, PELI	PELI2	OG5_129859, Pellino	Pellino	Pellino	Pellino	
**TAB1**	TrispH2_010884	TAB1	K04403, TAB1	TAB1	OG5_134489, TAB1	PPM-type phosphatase	PP2C	TAB1	
**TAK1 (MAP3K7)**	TrispH2_010261	MAP3K7	K04427, TAK1	MAP3K7	OG5_132679, TAK1	Tyrosine kinase	/	MAP3K7	
**Tollip**	TrispH2_005525	Tollip-a	K05402, Tollip	Tollip	OG5_134009, Tollip	C2, CUE	/	Tollip	
**TRAF**	TrispH2_006640	TRAF4	K09848, TRAF4	ENOG410V64Q, TRAF	OG5_131223, TRAF	Znf_RING, Znf_TRAF, MATH/TRAF	/	TRAF6	
	TrispH2_006642	TRAF4	K09848, TRAF4	ENOG410V64Q, TRAF	OG5_131223, TRAF	Znf_RING, Znf_TRAF, MATH/TRAF	TRAF4	TRAF6	
	TrispH2_008055	TRAF6-a	/	TRAF6	OG5_136539, TRAF	Znf_RING, TRAF-like, MATH/TRAF	TRAF6	TRAF4	

Additional References

1. Gerdol M, Venier P, Edomi P, Pallavicini A. Diversity and evolution of TIR-domain-containing proteins in bivalves and Metazoa: New insights from comparative genomics. Dev Comp Immunol. 2017;70:145–64. doi:10.1016/j.dci.2017.01.014.
