# Supplementary material for: Innate immunity in the simplest animals – placozoans
Source: BMC Genomics. 2019 Jan 5;20:5. doi: 10.1186/s12864-018-5377-3 (PMC6321704; doi:10.1186/s12864-018-5377-3)

TRAF – MATH/TRAF domain

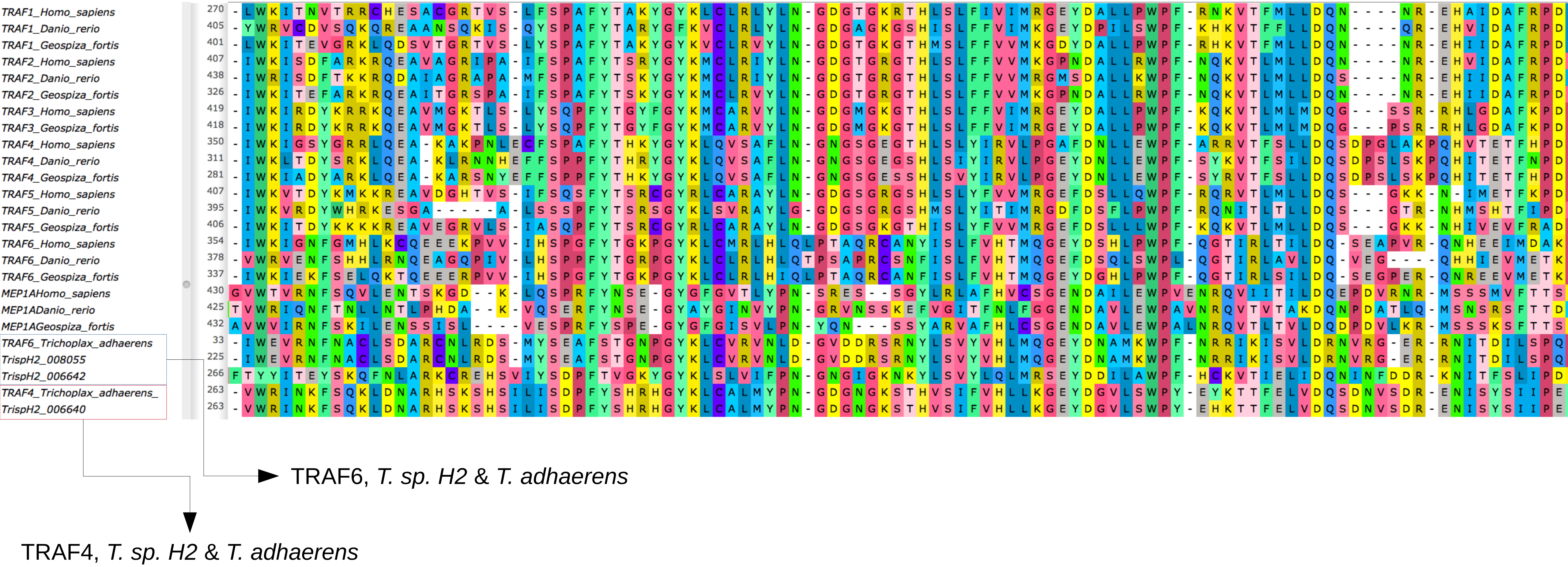

IRF- IRF domain (partial)

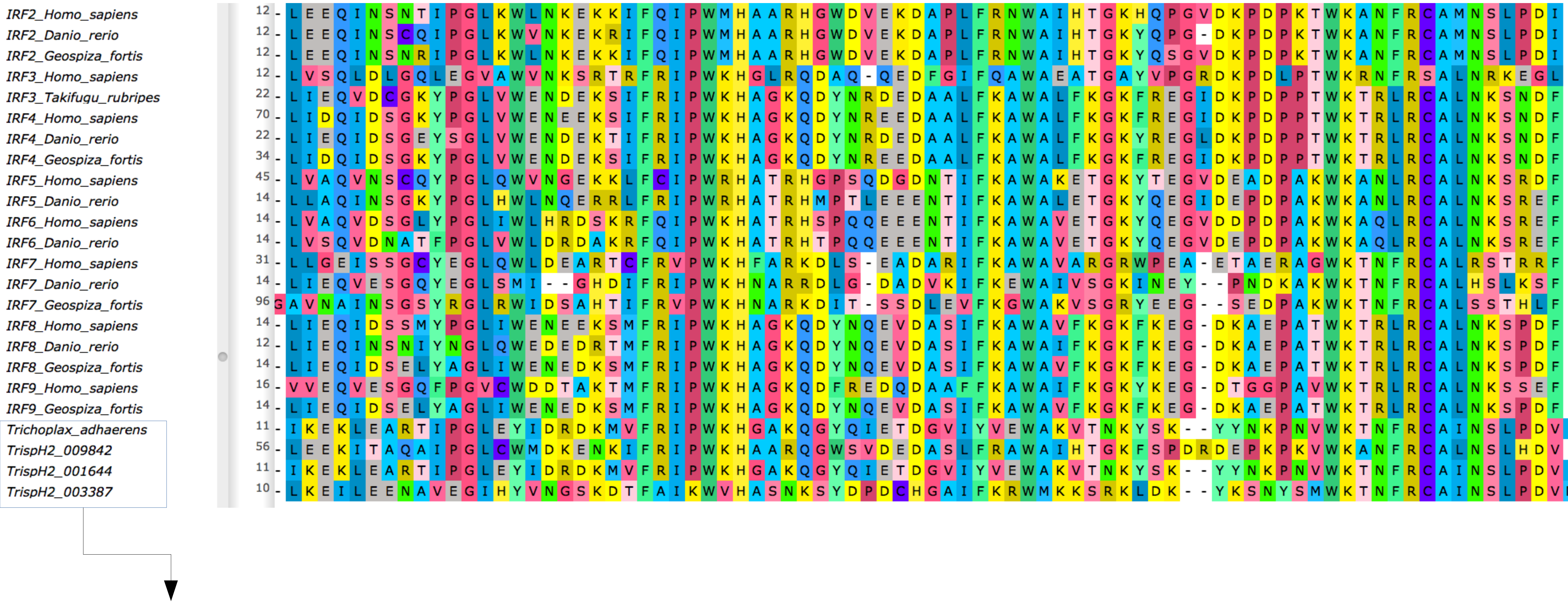

IRF, *T. sp. H2 & T. adhaerens*

IKK (Pkinase domain, partial)

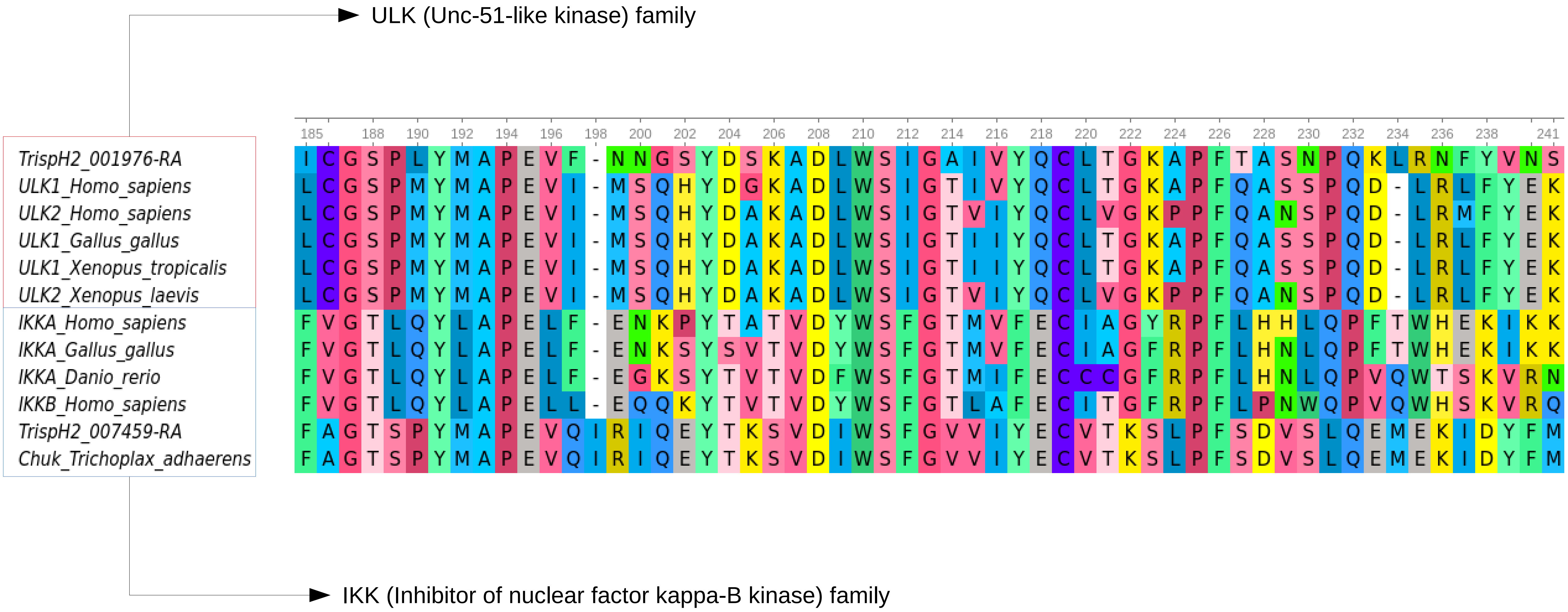

TAB1 (PP2C domain, partial)

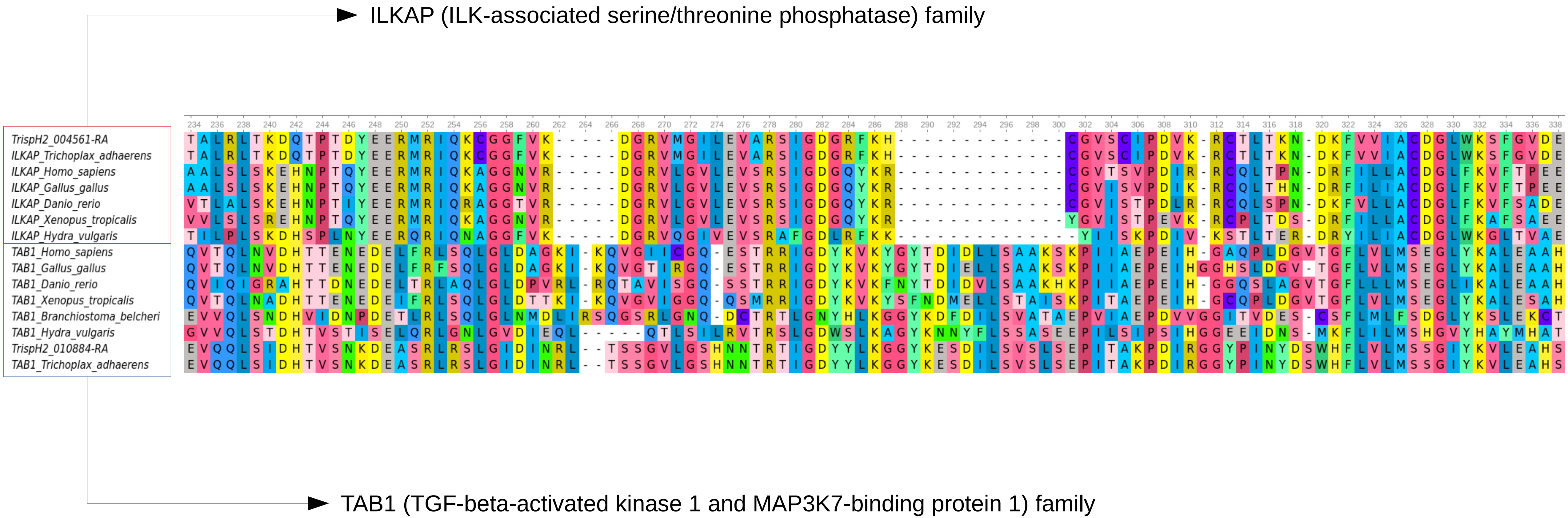

JUN/FOS (bZIP domain)

JUN_Homo_sapiens	253	KAERKRMRNRIAASKCRKRKLERIARLEEKVKTLKAQNSEL
JUN_Danio_rerio	224	KAERKRMRNRIAASKCRKRKLERISRLEDKVKTLKSQNSEL
JUN_Geospiza_fortis	234	KAERKRMRNRIAASKCRKRKLERIARLEEKVKTLKAQNSEL
FOS_Homo_sapiens	120	KRRIRRERNKMAAAKCRNRRRELTDTLQAETDQLEDEKSAL
FOS_Danio_rerio	95	KKRVRRERNKMAAAKCRNRRRELTDTLQAETDQLEDEKSAL
Trichoplax_adhaerens	179	KKERKRERNRLAASRCRKRKLEKEKDLQDRVKELKCENNKL
TrispH_000596	179	KKERKRERNRLAASRCRKRKLEKENDLQDRVKELKCENNKL

JUN, *T. sp. H2 & T. adhaerens*

MAP2K (Pkinase domain, partial)

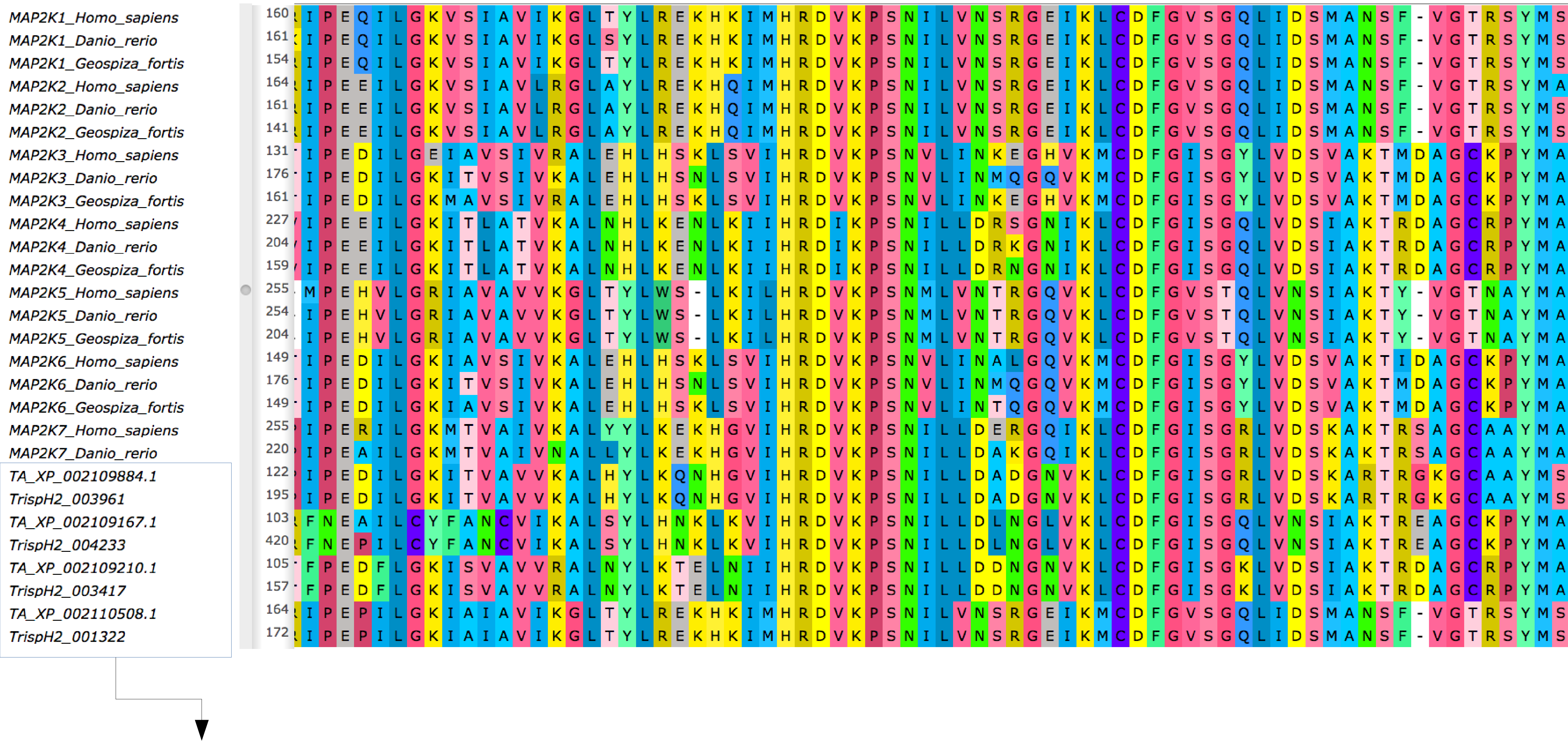

MAP2K, *T. sp. H2 & T. adhaerens*

MAP3K (Pkinase domain, partial)

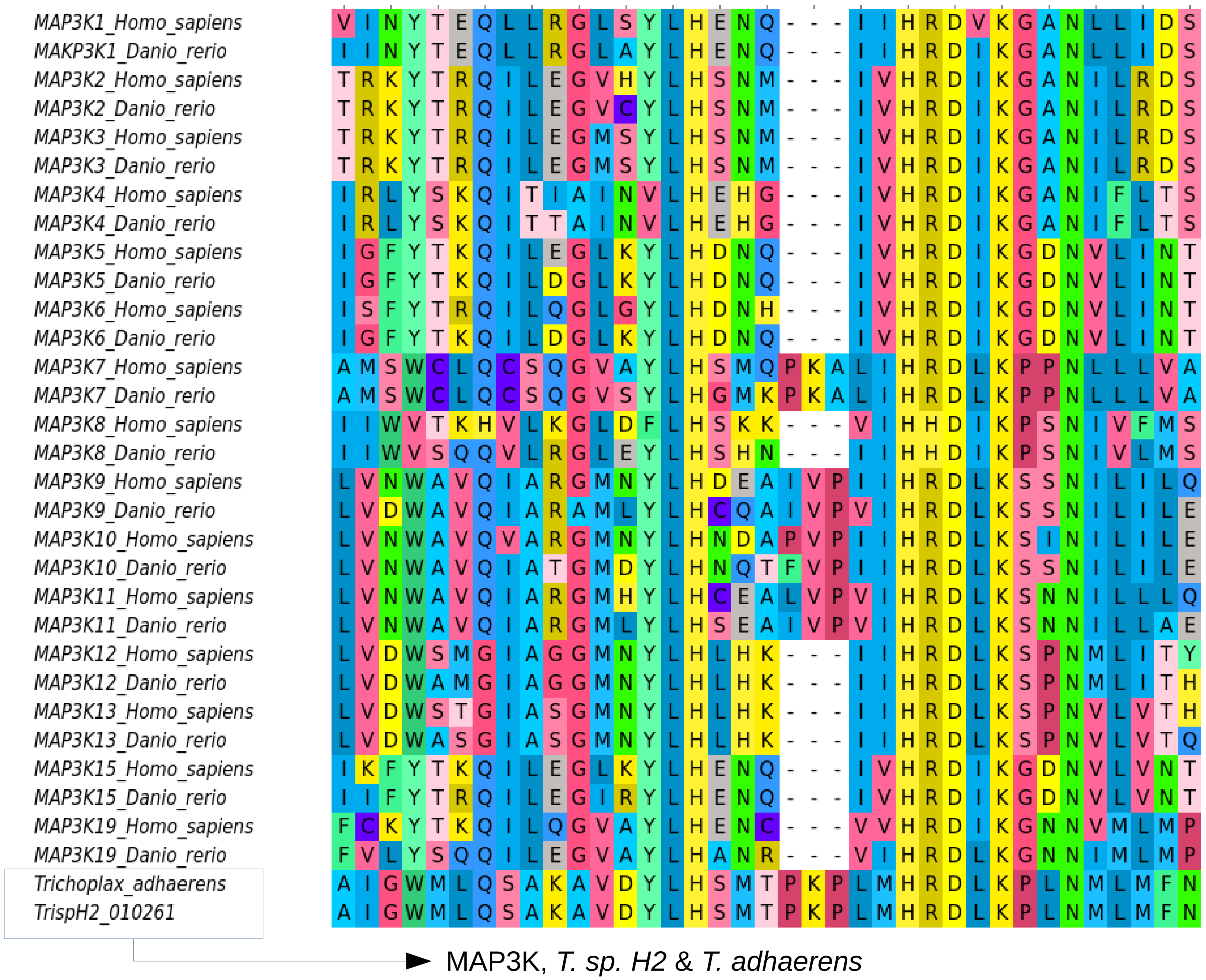

MAPK (Pkinase domain, partial)

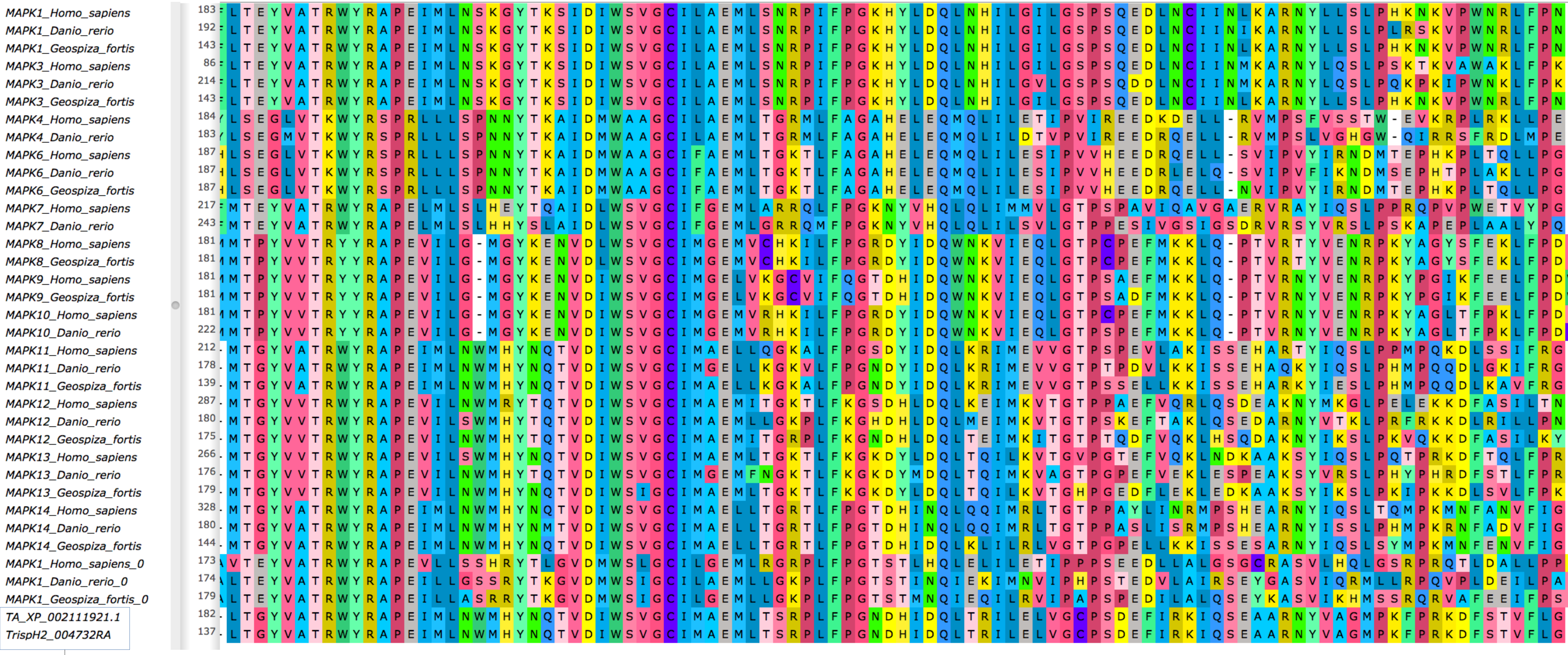

MAPK11/13/p38, *T. sp. H2 & T. adhaerens*

NFKB – Ankyrin repeats

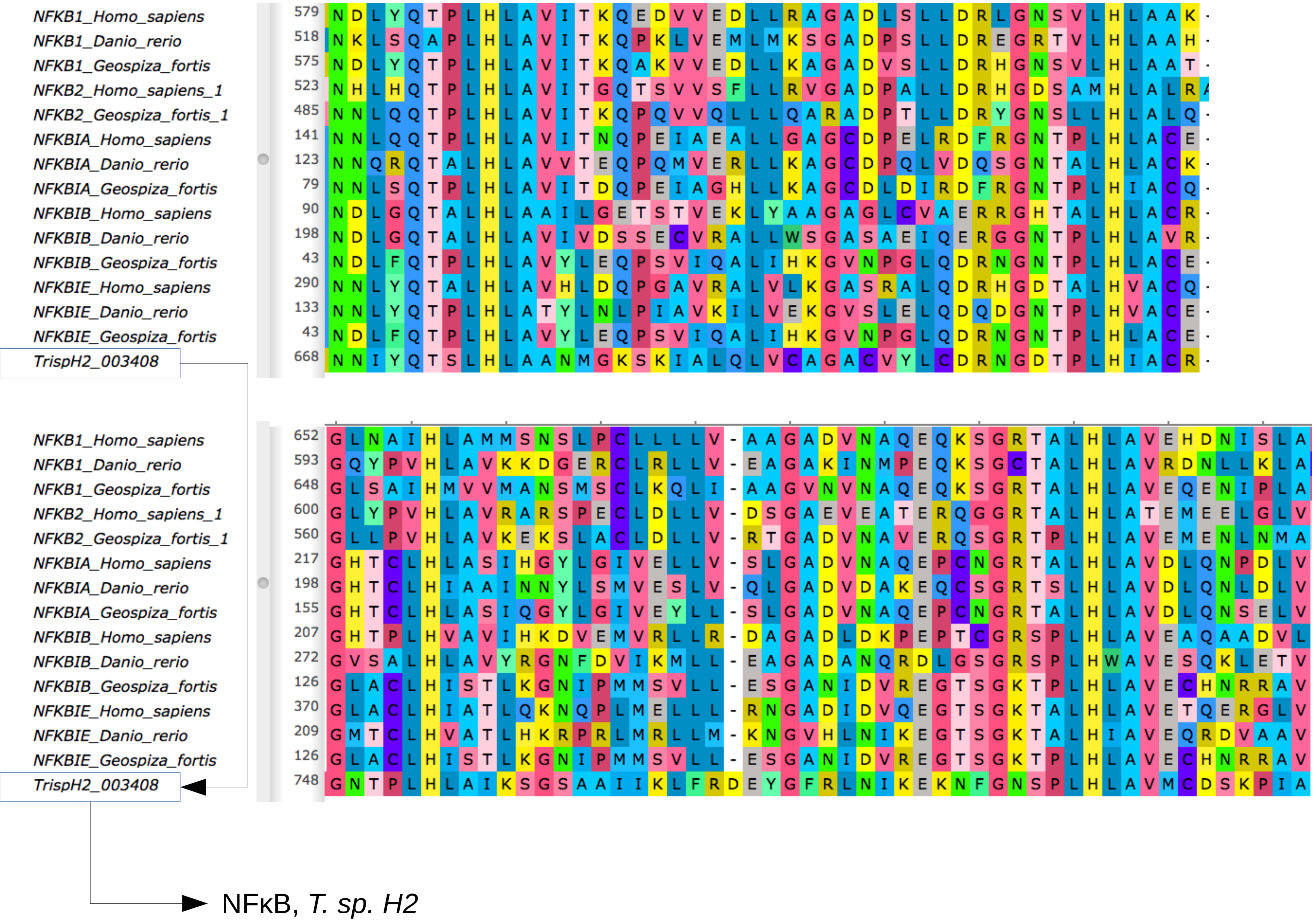

Tollip, MCTP (C2 domain)

3rd C2 domain of MCTP2 *D.rerio* & *H.sapiens* & 2nd C2 domain of MCTP1 *Trichoplax sp. H2*

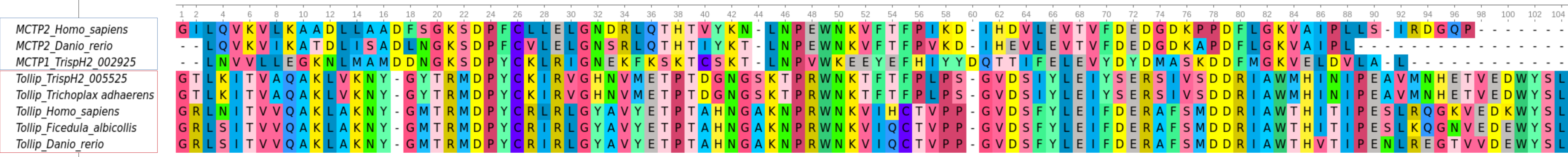

Tollip, *T. sp. H2* & *T. adhaerens*, C2 domain

Pellino domain (amino end)

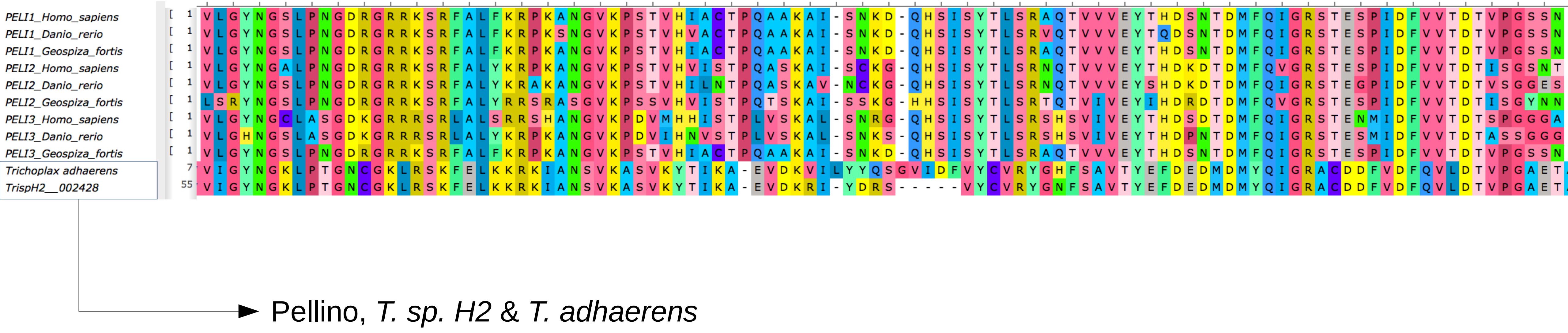

IRAK – Pkinase domain, carboxy end

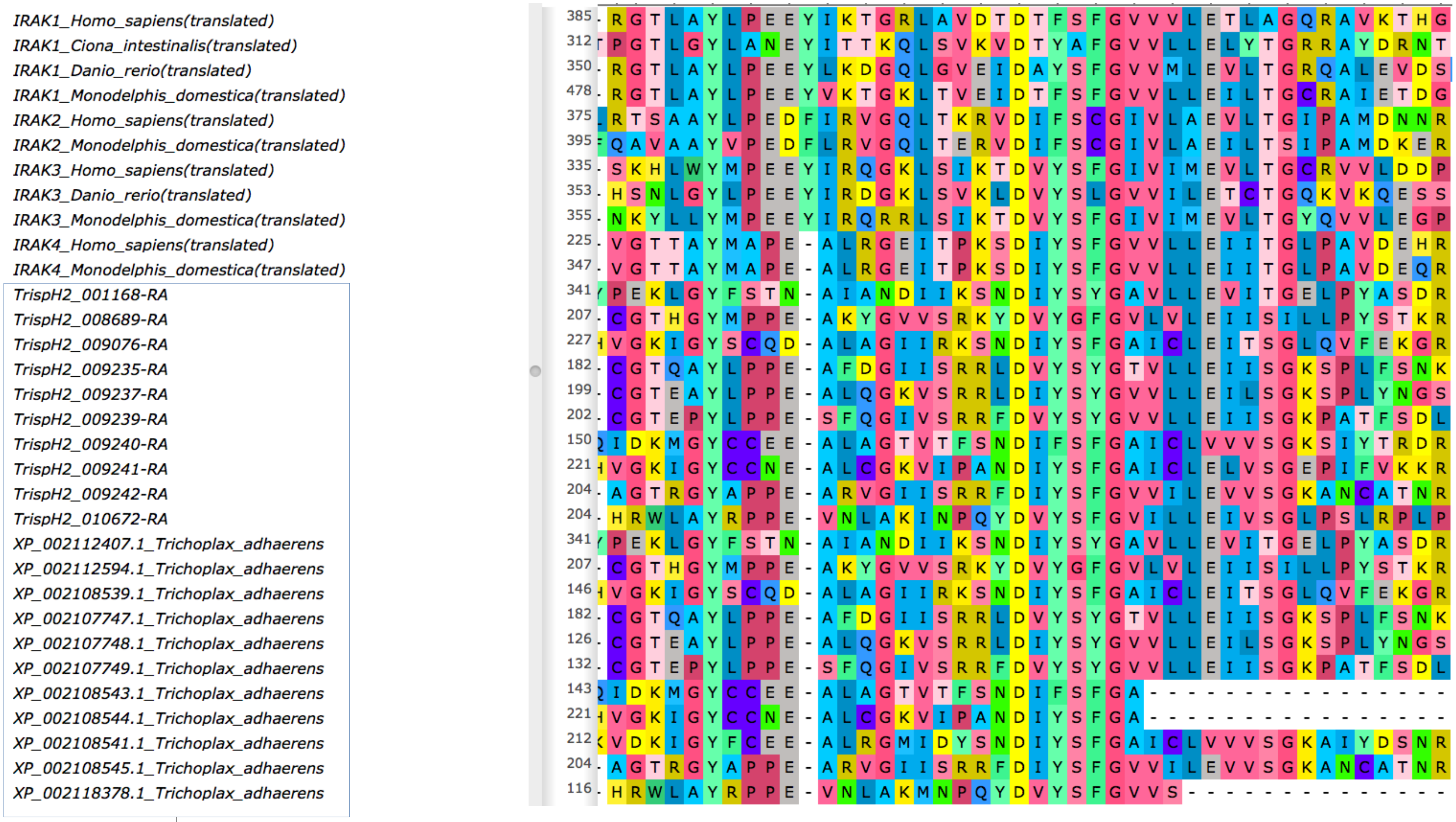

Examples of IRAK-like genes in *T. sp. H2* & *T. adhaerens*

DUSP family (DSPc domain, partial)

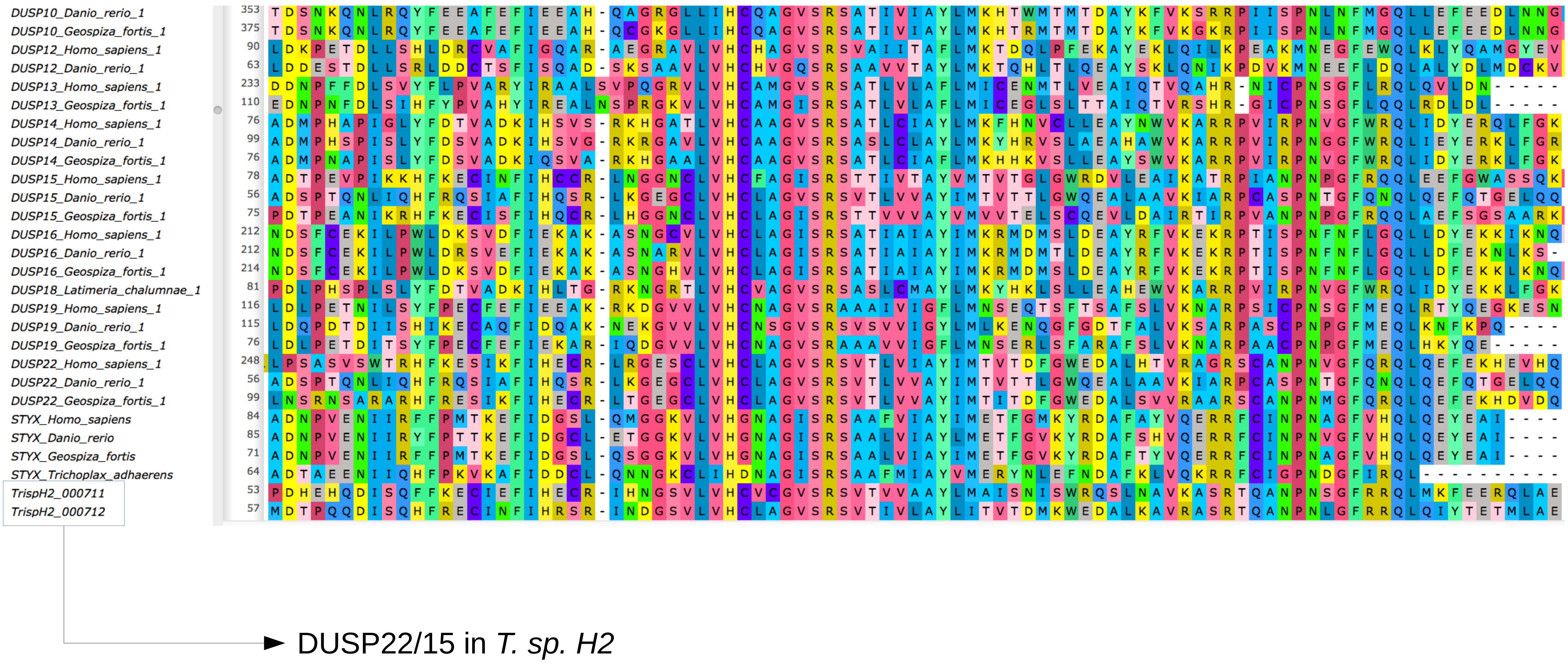

Supplement: Supplementary file 3 — Dataset S2. Conserved domain alignments of the TLR pathway genes. (PDF 2446 kb) [file 12864_2018_5377_MOESM3_ESM.pdf]
