# Supplementary material for: Innate immunity in the simplest animals – placozoans
Source: BMC Genomics. 2019 Jan 5;20:5. doi: 10.1186/s12864-018-5377-3 (PMC6321704; doi:10.1186/s12864-018-5377-3)
Supplement: Supplementary file 5 — Figure S2. Vertebrate and placozoan intelectins aligned in the region of the fibrinogen-related domain (FReD) of placozoan intelectins. (PDF 117 kb) [file 12864_2018_5377_MOESM5_ESM.pdf]

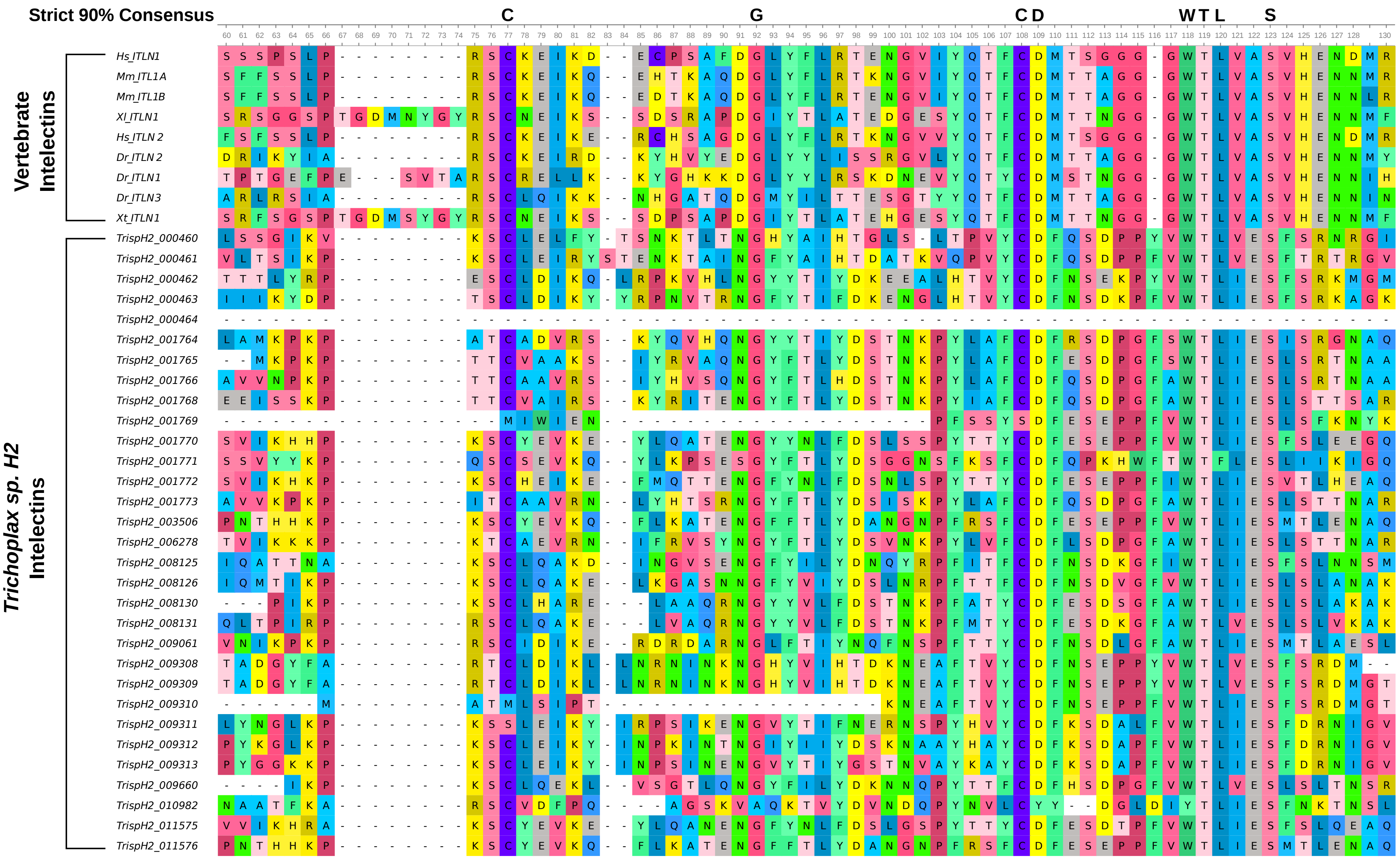

Strict 90% Consensus
C G C D W T L S
Vertebrate Intelectins
Hs_ITLN1
Mm_ITL1A
Mm_ITL1B
Xl_ITLN1
Hs_ITLN 2
Dr_ITLN 2
Dr_ITLN1
Dr_ITLN3
Xt_ITLN1
Trichoplax sp. H2 Intelectins
TrispH2_000460
TrispH2_000461
TrispH2_000462
TrispH2_000463
TrispH2_000464
TrispH2_001764
TrispH2_001765
TrispH2_001766
TrispH2_001768
TrispH2_001769
TrispH2_001770
TrispH2_001771
TrispH2_001772
TrispH2_001773
TrispH2_003506
TrispH2_006278
TrispH2_008125
TrispH2_008126
TrispH2_008130
TrispH2_008131
TrispH2_009061
TrispH2_009308
TrispH2_009309
TrispH2_009310
TrispH2_009311
TrispH2_009312
TrispH2_009313
TrispH2_009660
TrispH2_010982
TrispH2_011575
TrispH2_011576

Figure S2 Vertebrate and placozoan intelectins aligned in the region of the fibrinogen-related domain (FReD) of placozoan intelectins. The identified FReDs in placozoan intelectins are shorter than in vertebrate intelectins, probably due to the divergence from canonical domain models, and restricted to the N-terminal portion of the proteins. Only two of the ten conserved cysteine residues in placozoan intelectins are contained in this part. Highly conserved residues are indicated by a strict 90% consensus at the top. Note that TrispH2_000464 is truncated and contains no sequence information in this part of the alignment. See also Additional file 4: Dataset S3C and for the complete alignment Additional file 6: Dataset S4.
