# Supplementary material for: Innate immunity in the simplest animals – placozoans
Source: BMC Genomics. 2019 Jan 5;20:5. doi: 10.1186/s12864-018-5377-3 (PMC6321704; doi:10.1186/s12864-018-5377-3)

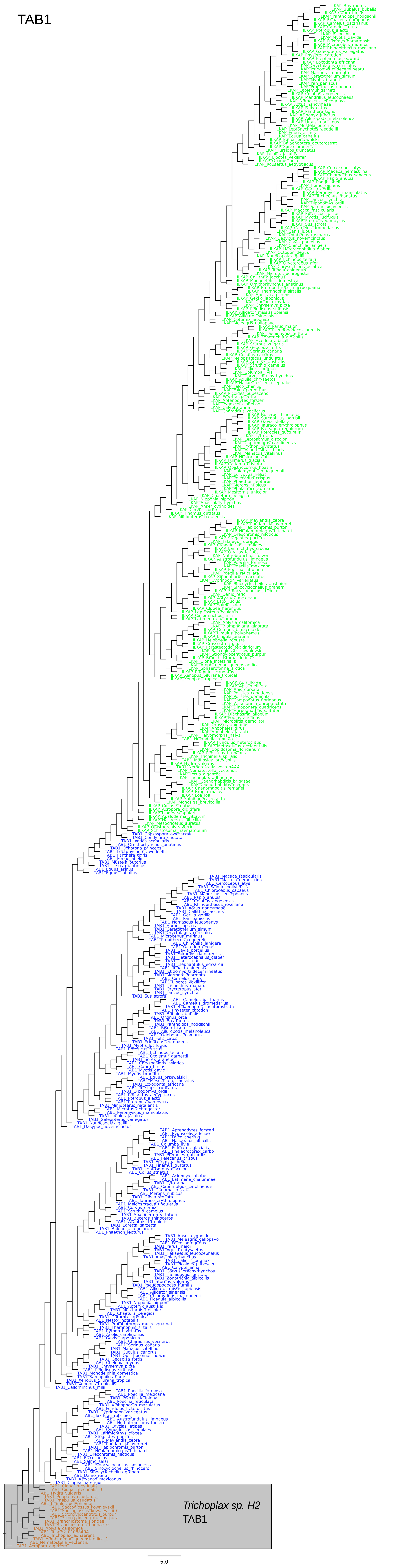
TAB1
Trichoplax sp. H2
TAB1
6.0

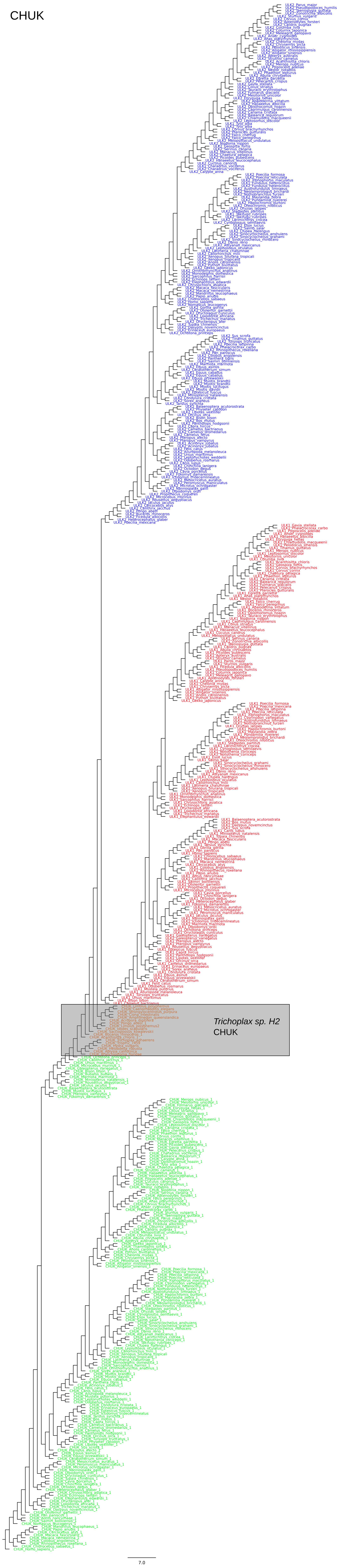
CHUK
Trichoplax sp. H2
CHUK
7.0

JSP/DUSP22 (partial)

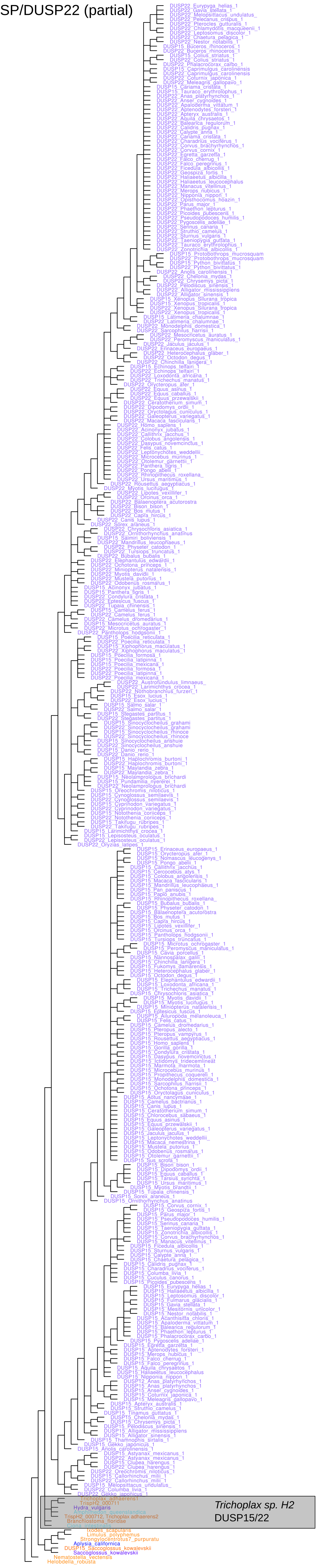

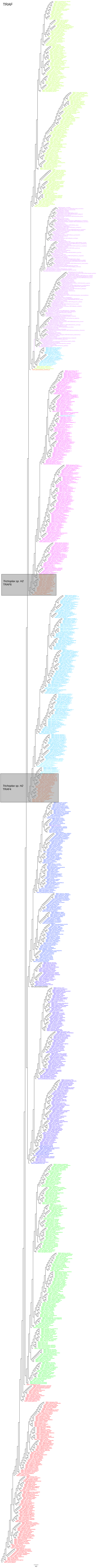
TRAF
Trichoplax sp. H2
TRAF6
Trichoplax sp. H2
TRAF4

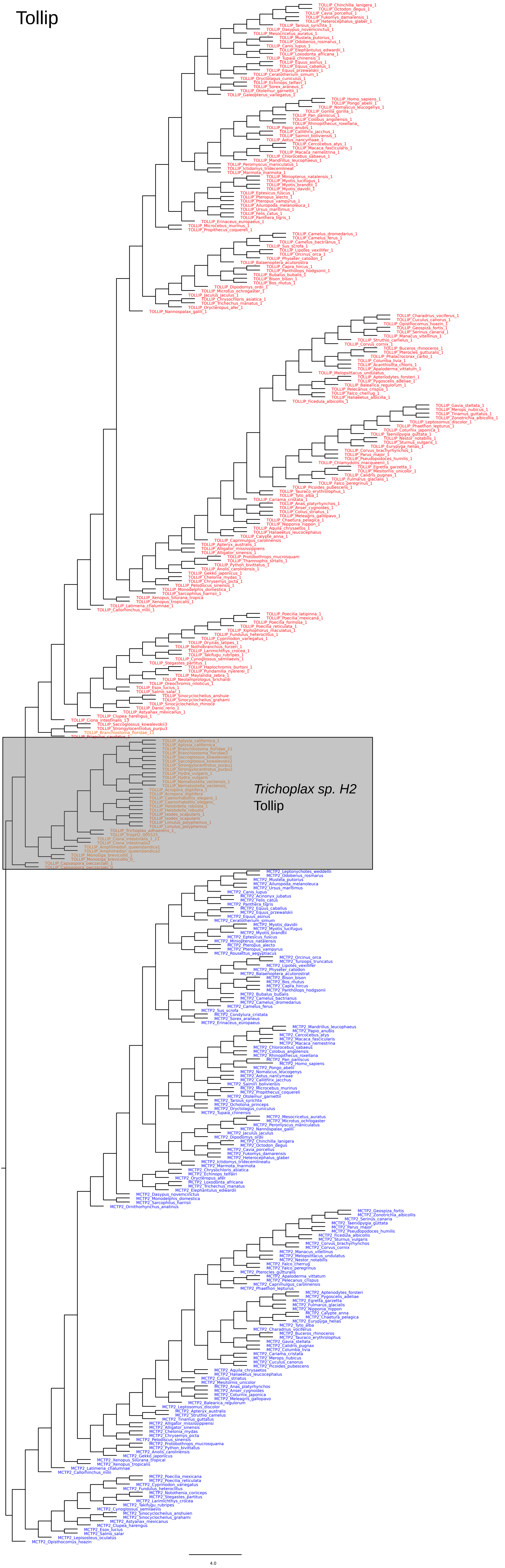
Tollip
Trichoplax sp. H2
Tollip
4.0

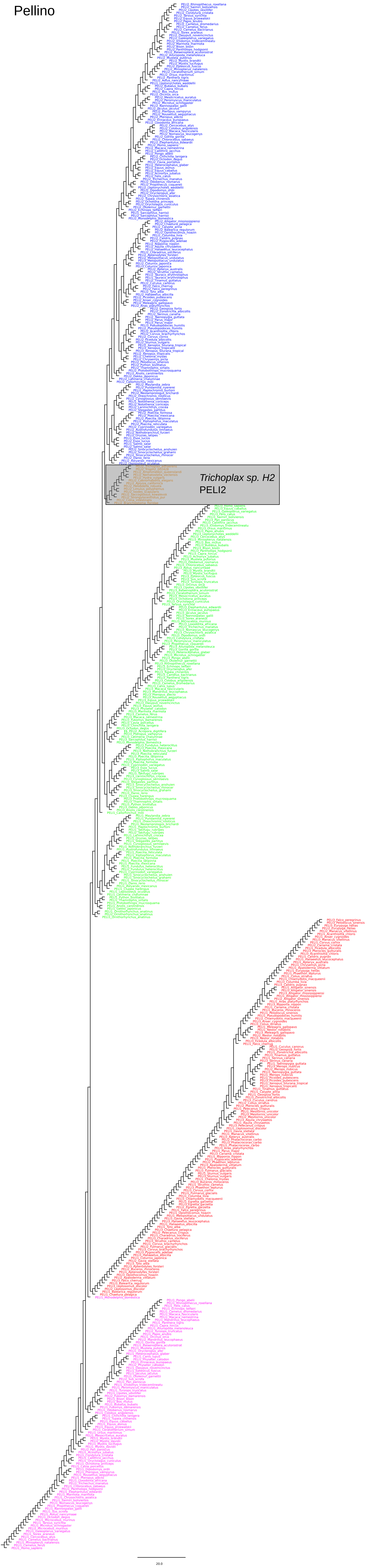
Pellino
Trichoplax sp. H2
PELI2
20.0

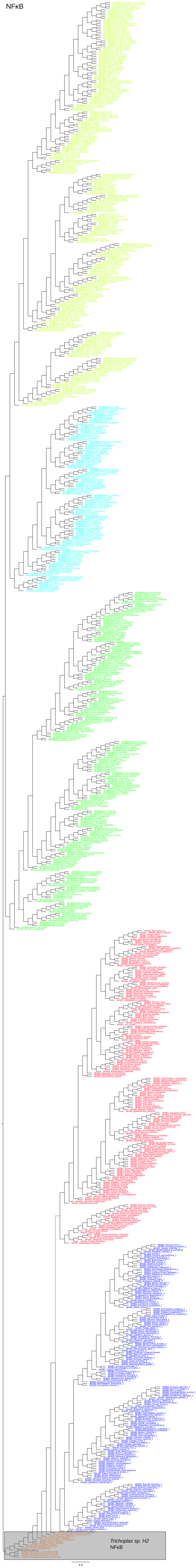
NFκB
Trichoplax sp. H2
NFκB
4.0

MAPK

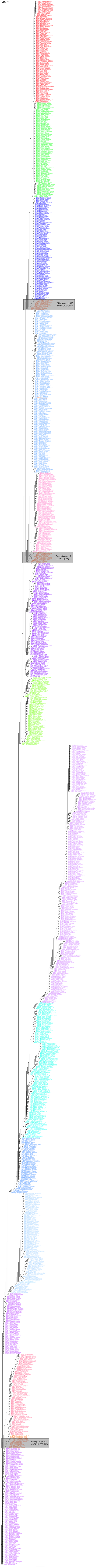
Trichoplax sp. H2
MAPK9/10 (JNK)
Trichoplax sp. H2
MAPK11 (p38)
Trichoplax sp. H2
MAPK1/3 (ERK1/3)

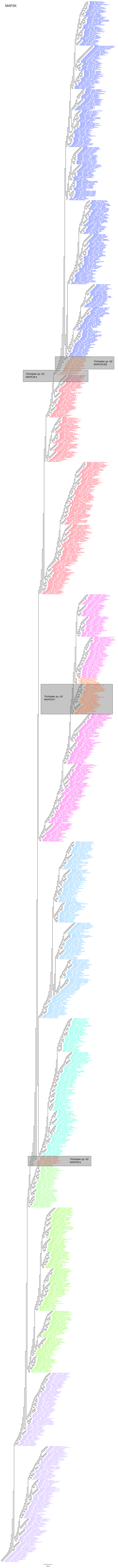
MAP2K
Trichoplax sp. H2
MAPK2K3/6
Trichoplax sp. H2
MAPK2K4
Trichoplax sp. H2
MAPK2K7
Trichoplax sp. H2
MAPK2K1
10.0

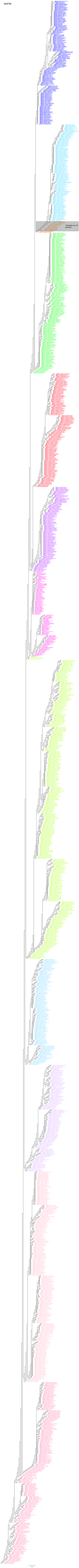
MAP3K
Trichoplax sp. H2
MAP3K7
8.0

JUN/FOS

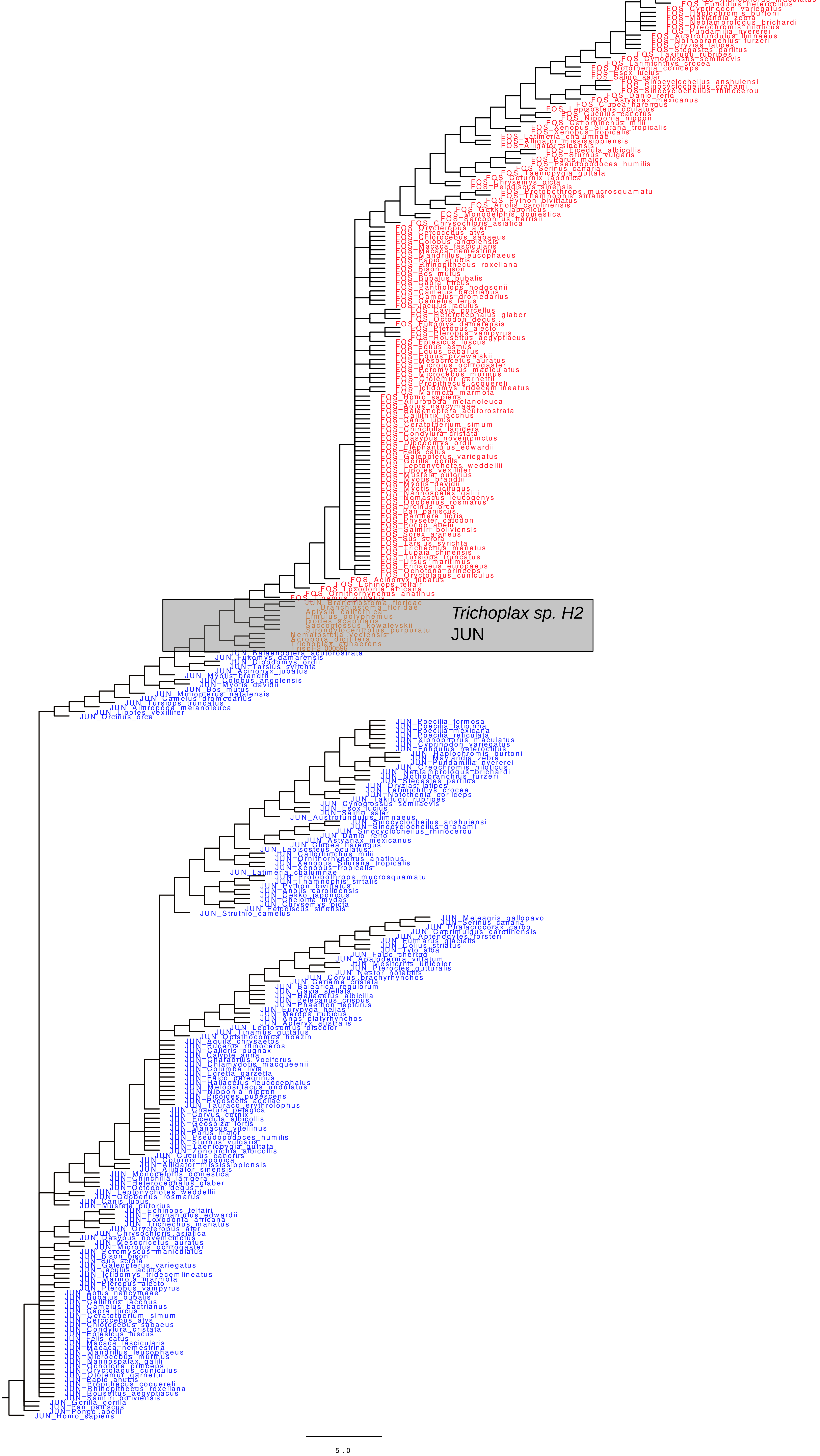

IRF

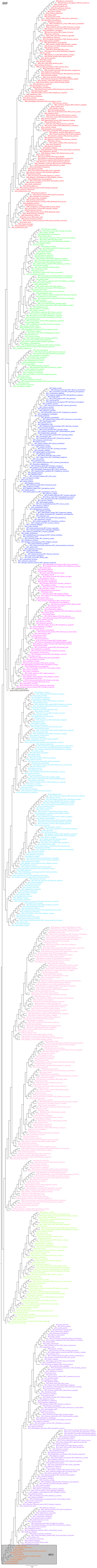

IRAK

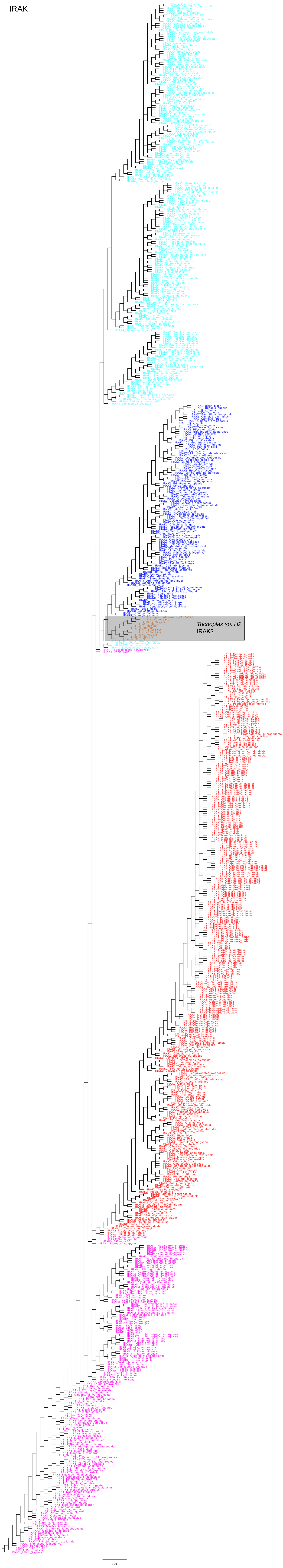

Supplement: Supplementary file 2 — Dataset S1. Trees of the phylogenetic validation of the TLR pathway genes. (PDF 1122 kb) [file 12864_2018_5377_MOESM2_ESM.pdf]
